# Risk factors for voluntary early old-age retirement in middle-aged workers: A meta-analysis

**DOI:** 10.5271/sjweh.4241

**Published:** 2025-11-01

**Authors:** Rahman Shiri, Joonas Poutanen, Eija Haukka, Mikko Härmä, Jenni Ervasti

**Affiliations:** 1 Finnish Institute of Occupational Health, Helsinki, Finland.

**Keywords:** depression, employment, health status, mental health, occupational exposure

## Abstract

**Objective:**

This meta-analysis aimed to identify sociodemographic, lifestyle, work-related and health risk factors for voluntary early old-age retirement among middle-aged workers.

**Methods:**

Searches were conducted in PubMed, Web of Science, PsycInfo, and Scopus from their inception until February 2025. Observational longitudinal studies involving workers aged 40–64 years were included. Two reviewers evaluated the methodological quality of the studies. A random-effects meta-analysis was performed, and heterogeneity and publication bias were assessed.

**Results:**

From 13 899 publications, 23 longitudinal studies (N=2 270 430 participants) were included. The following factors were associated with an increased risk of early old-age retirement: age [hazard ratio (HR) 1.35, 95% CI (confidence interval) 1.12–1.63 per year increase], overweight or obesity (HR 1.10, 95% CI 1.03–1.17), physically demanding work (HR 1.29, 95% CI 1.05–1.59), low job control (HR 1.14, 95% CI 1.11–1.17), low influence at work (HR 1.10, 95% CI 1.02–1.19), low organizational justice (HR 1.27, 95% CI 1.10–1.46), lack of skills and knowledge development (HR 2.16, 95% CI 1.63–2.85), suboptimal self-rated general health (HR 1.22, 95% CI 1.12–1.34), chronic physical conditions (HR 1.11, 95% CI 1.05–1.17), and depressive symptoms (HR 1.34, 95% CI 1.12–1.61). Conversely, a lower risk was found among individuals who were unmarried, separated, or widowed (HR 0.74, 95% CI 0.60–0.91).

**Conclusions:**

This meta-analysis underscores the impact of overweight, physical and psychosocial work factors, lacking skills and knowledge development and health conditions on early old-age retirement risk among middle-aged workers. Targeted interventions to encourage healthy lifestyles, foster a supportive work environment, and promote mental health may help to reduce early old-age retirement risk.

With a growing elderly population due to increased life expectancy and declining birth rates, the retirement age has been progressively rising across the Organisation for Economic Cooperation and Development (OECD) countries (1, 2). Concurrently, the approval criteria for disability retirement have become more stringent (1, 2). As a result, there has been a surge in the number of individuals opting for voluntary early old-age retirement, a trend that may have disproportionately impacted blue- compared to white-collar workers (1). To address these challenges and alleviate financial pressures on pension systems, several countries, particularly within the OECD, are shifting from voluntary early old-age retirement to part-time retirement options to extend working lives (2). Voluntary early old-age retirement is a common pathway out of working life, with unemployment and disability retirement being other common exit routes (3–5).

Worldwide, there has been an increase in health risk factors such as obesity, sedentary lifestyles, and chronic conditions like diabetes, mental health conditions, and musculoskeletal disorders (6, 7). This rise in health concerns is linked to higher rates of early retirement or unemployment (5). In addition to health risks, previous research has shown that age (4, 8) and certain occupational factors – such as low influence at work, low levels of social capital and low organizational justice (9, 10) – are associated with the increased rates of voluntary early old-age retirement. However, previous studies have reported contradictory results regarding the role of sex (4, 11), education (8, 11) and occupational class (8, 12) in voluntary early old-age retirement. The risk is higher for individuals who have the financial means to stop working before the retirement age and for those whose partners have a positive attitude toward early retirement (13).

While some risk factors for voluntary early old-age retirement among middle-aged workers have been identified, there remains a significant gap in comprehensive information regarding these factors. To effectively extend working careers of aging workers, it is crucial to pinpoint both individual and work-related risk factors that contribute to voluntary early old-age retirement. This understanding is essential for developing targeted preventive interventions and programs. In our current study, we aimed to address this gap by conducting a systematic review and meta-analysis of observational longitudinal studies. Our objective was to estimate the magnitudes of the effects of various sociodemographic, lifestyle, occupational factors, and health conditions on the risk of voluntary early old-age retirement among middle-aged individuals.

## Methods

### Search strategy

Our review protocol and meta-analysis were developed in accordance with the PRISMA statement (14). We conducted a comprehensive search of the PubMed, Web of Science, PsycInfo, and Scopus databases from their inception until February 2025, using a combination of MeSH terms and text words as detailed in table 1. Within the PI(E)COTS framework, we focused on ‘P’ (population) to limit our search to middle-aged workers and ‘O’ (outcome) to create a sensitive search string, ensuring no relevant studies were overlooked. We did not use ‘I(E)’ (intervention/exposure for observational studies), ‘C’ (control/comparison group), or ‘S’ (setting) in our search string. We applied a filter for ‘T’ (types of studies) to exclude clinical trials and reviews but did not restrict the search by publication year (15). Additionally, we extended our search to include Google Scholar and manually searched the reference lists of included reports and seven previous systematic reviews on disability retirement (16–22).

**Table 1:** Searches performed on PubMed, Web of Science, and Scopus on 12 February 2025.

Search	Query	Items found (N)
**PubMed**		
#1	“Unemployment”[Mesh] OR “disability pension”[tiab] OR “disability retirement”[tiab] OR “early retirement”[tiab] OR “retired early”[tiab] OR “labor market exit”[tiab] OR “labour market exit”[tiab] OR “personnel turnover”[Mesh] OR (quit[tiab] AND job[tiab]) OR (quit[tiab] AND organization[tiab]) OR (quit[tiab] AND organisation[tiab]) OR (quit[tiab] AND employment[tiab])	18 447
#2	“Middle aged”[Mesh] OR “old people”[tiab] OR “older people”[tiab] OR “old individuals”[tiab] OR “older individuals”[tiab] OR “old participants”[tiab] OR “older participants”[tiab] OR “old workers”[tiab] OR “older workers”[tiab]OR “40 years”[tiab] OR “45 years”[tiab] OR “50 years”[tiab] OR “55 years”[tiab] OR “60 years”[tiab]	5 133 746
#3	#1 AND #2	6656
#4	clinical trials and reviews	308
#5	#3 NOT #4	6348
**Web of Science**		
#1	ALL=(unemploy*) OR ALL=(“disability pension”) OR ALL=(“disability retirement”) OR ALL=(“early retire*”) OR ALL=(“retired early”) OR ALL=(“labor market exit”) OR ALL=(“labour market exit”) OR ALL=(“personnel turnover”) OR ALL=(quit AND job) OR ALL=(quit AND organization) OR ALL=(quit AND organisation) OR ALL=(quit AND employment)	73 204
#2	ALL=(“middle aged”) OR ALL=(“middle age”) OR ALL=(“old people”) OR ALL=(“older people”) OR ALL=(“old individuals”) OR ALL=(“older individuals”) OR ALL=(“old participants”) OR ALL=(“older participants”) OR ALL=(“old workers”) OR ALL=(“older workers”) OR ALL=(“40 years”) OR ALL=(“45 years”) OR ALL=(“50 years”) OR ALL=(“55 years”) OR ALL=(“60 years”)	496 445
#3	#1 AND #2	3414
#4	reviews, proceeding abstracts, meeting abstracts, letters, and editorials	164
#5	#3 NOT #4	3250
**Scopus**		
#1	TITLE-ABS-KEY (unemployment OR “disability pension” OR “disability retirement” OR “early retirement” OR “retired early” OR “labor market exit” OR “labour market exit” OR “personnel turnover” OR “quit job” OR “quit organization” OR “quit organisation” OR “quit employment”)	95 230
#2	TITLE-ABS (“Middle aged” OR “old people” OR “older people” OR “old individuals” OR “older individuals” OR “old participants” OR “older participants” OR “old workers” OR “older workers” OR “40 years” OR “45 years” OR “50 years” OR “55 years” OR “60 years”)	658 106
#3	#1 AND #2	3315
#4	Limited to articles and English	2655

### Inclusion and exclusion criteria

This review included longitudinal studies focusing on middle-aged employees, specifically those aged 45–64 years. Recognizing that many studies targeted individuals aged ≥40, we expanded our inclusion criteria to encompass studies involving workers aged 40–64 years. These studies needed to examine a risk factor related to voluntary early old-age retirement, disability retirement, unemployment, or job change. In this review, we focus exclusively on voluntary early old-age retirement, defined as retirement before the country-specific minimum official retirement age (13, 23, 24). We excluded research involving clinical subjects, individuals who were exclusively unemployed, solely on sick leave, or already retired. Furthermore, studies that combined different forms of labor market exits into a single outcome, such as combining voluntary early old-age retirement with unemployment or disability retirement, were also excluded.

### Quality assessment

Two reviewers independently assessed the methodological quality of the studies using criteria adapted from the Effective Public Health Practice Project quality assessment tool (supplementary material, www.sjweh.fi/article/4241, table S1) (25). The evaluation focused on five key potential biases: selection, performance, detection, attrition, and confounding, as detailed in supplementary figures S1–S2. Any disagreements between the reviewers were resolved through discussion.

### Risk factors

The risk factors included sociodemographic factors, lifestyle habits, occupational factors, and health conditions. The sociodemographic factors included age, sex, marital status, education, and occupational class. Lifestyle factors encompassed smoking, body mass index (BMI), leisure-time physical activity, and excessive alcohol consumption. Occupational factors comprised physical and psychosocial factors at work, as well as opportunities for skills and knowledge development. Health conditions ranged from self-rated general health to daily activity limitations and included both physical and mental conditions.

### Voluntary early old-age retirement

Voluntary early old-age retirement involves choosing to leave the workforce before reaching the official retirement age, either partially or fully. This decision is often supported by programs like the Voluntary Retirement Scheme (eg, in some Nordic countries), which provide employees with the option to retire early and receive benefits (13). The Voluntary Retirement Scheme requires a minimum retirement age, which is the earliest age at which an individual can opt to retire and start receiving retirement benefits. This age varies by country and is influenced by factors such as national policies, economic conditions, and life expectancy (9, 13, 23, 26). Voluntary early old-age retirement is not based on health conditions and can also be applied to individuals who do not qualify for disability retirement (12). In many countries, this type of retirement is available to individuals with paid employment starting at age 61 (12, 23). However, programs like the Voluntary Retirement Scheme allow for earlier retirement under specific conditions, providing greater flexibility for employees.

### Statistical analysis

When encountering multiple reports from a single study, we prioritized extracting data from those that provided more thorough adjustments for confounding variables or included a larger sample size. For each study, we obtained the most adjusted risk estimates and their 95% confidence intervals (CI) for the exposures. Two studies (4 reports) (23, 27–29) presented findings for two outcomes: voluntary early old-age retirement without income and voluntary early old-age retirement with income. Another study (30) provided results for voluntary early old-age retirement before age 55 and for voluntary early old-age retirement at ≥55 years. For these studies, we included voluntary early old-age retirement with income and voluntary early old-age retirement at age ≥55 in the meta-analyses.

Three studies compared the risk of voluntary early old-age retirement for men with that for women (4, 8, 11). To combine these studies with others that compared the risk for women with that for men, we inverted the men’s hazard ratio (HR) to derive the HR for women and used the standard error of the men’s estimate to calculate the 95% CI. To examine the effects of education and occupational class on voluntary early old-age retirement, we recalculated the adjusted HR by comparing workers with ≤12 years of education with those with >12 years of education (4, 8, 11, 12, 27, 31, 32) and workers in intermediate and low occupational classes with those in high occupational class (8, 12, 33). Another study (10) compared past and never smokers with current smokers. To compare past and current smokers with never smokers, we first inverted the HR for never smokers to obtain the HR for current smokers and divided the HR for past smokers by that of never smokers. We then used the standard error of the never smokers’ estimate to calculate the 95% CI for current smokers. A different study (3) compared lack of physical activity with moderate or vigorous physical activity. To combine this study with another (10) that compared moderate or vigorous physical activity with lack of physical activity, we inverted the HR for lack of physical activity to obtain the HR for moderate or vigorous physical activity and used the standard error of the lack of physical activity estimate to calculate the 95% CI.

One study (11) compared healthy workers with those diagnosed with a chronic physical condition. To estimate the effect of a chronic physical condition on the likelihood of voluntary early old-age retirement, we inverted the estimate for healthy workers and used the average standard errors of chronic obstructive pulmonary disease, heart failure, and diabetes to calculate 95% CI. Additionally, logistic regression was employed to identify risk factors for voluntary early old-age retirement in this study. We converted the odds ratios and their 95% CI to risk ratios, noting that 22.5% of the participants retired early during the follow-up period. We used a fixed-effect meta-analysis to integrate subgroups within a single study and a restricted maximum likelihood (REML) random-effects meta-analysis to synthesize findings from various studies (34). Meta-regression was employed to explore sources of variability in effect sizes across different studies (34). A funnel plot was used to examine publication bias, and Egger’s regression test was used to assess the asymmetry of the funnel plot (34). Additionally, the trim-and-fill method was applied to impute potentially missing studies due to publication bias (34). Borderline statistical significance was defined as a P >0.05 but <0.10. Stata version 18, (StataCorp, College Station, TX, USA) was used for conducting the meta-analyses.

## Results

**Figure 1:**
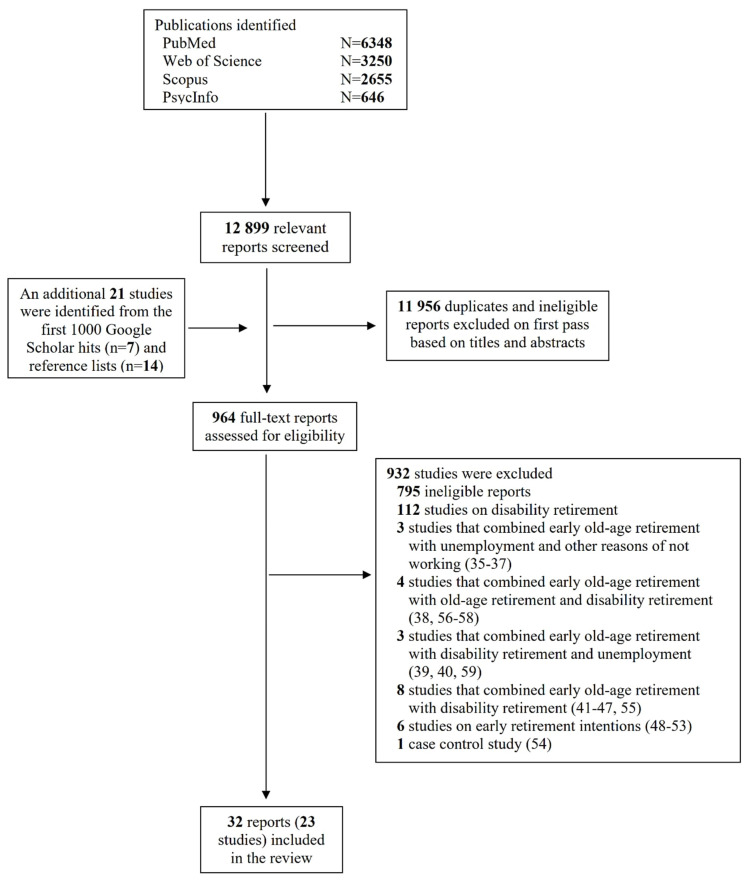
PRISMA flow diagram of the studies selection.

A total of 12 899 reports were collected from PubMed, Web of Science, PsycInfo and Scopus, as shown in figure 1. The first reviewer screened the titles and abstracts, including the first 1000 results from Google Scholar, bringing the total to 13 899. Of these, 12 935 duplicates and ineligible publications were excluded. Subsequently, 964 articles were examined in full to assess their relevance, with most of these full-text articles screened to determine the age of the participants, which was not reported in the abstracts. Of these, 907 were excluded for not studying voluntary early old-age retirement and 25 studies (35–59) failed to meet the inclusion criteria. This review ultimately encompassed 32 reports (3–5, 8–13, 23, 24, 26–33, 60–72), which included 23 longitudinal studies involving 2 270 430 participants. The results of seven studies were detailed across 19 distinct reports, while the findings from four studies were consolidated into a single report. The number of participants in the studies ranged from 1167 to 1 000 000 individuals (supplementary table S2). The distribution of the 23 studies includes seven from Denmark, six from the Netherlands, three from Sweden, two from Norway, and one each from Finland, France, Germany, and the United Kingdom. Additionally, one study included participants from 11 different European countries: Austria, Belgium, Denmark, France, Germany, Greece, Italy, Spain, Sweden, Switzerland, and the Netherlands.

### Methodological quality of the studies

Six studies were assessed as having a low risk of selection bias, twelve were rated as moderate risk, and five were identified as high risk, as detailed in supplementary figure S1. For performance bias, three studies were rated as low risk, while nineteen studies were considered moderate risk, as shown in supplementary figures S1–S2. Additionally, one study (3 reports) was considered to have a low risk of bias for some exposures (23, 29) and a moderate risk for another exposure (28). Regarding detection bias, fourteen studies were evaluated as low risk, and nine as moderate risk. In terms of confounding, six studies were deemed to have a low risk of bias, having accounted for most confounding factors. Fourteen studies were rated as having a moderate risk of bias, as they only adjusted for some confounders in their observed associations, and one study (32) was rated as having a high risk. Additionally, two studies (six reports) were considered to have a low risk of bias for some exposures (3, 64) and a moderate risk for others (5, 63, 68, 69). One study was rated to have a moderate risk for one exposure (23) and a high risk for other exposures (28, 29). Concerning attrition bias, fifteen studies were considered low risk, six moderate risk, and one study was marked as high risk. Furthermore, one study (four reports) was rated as having a low risk of attrition bias for one report (13), but a moderate risk for other reports (4, 24, 67).

### Meta-analysis

**Figure 2:**
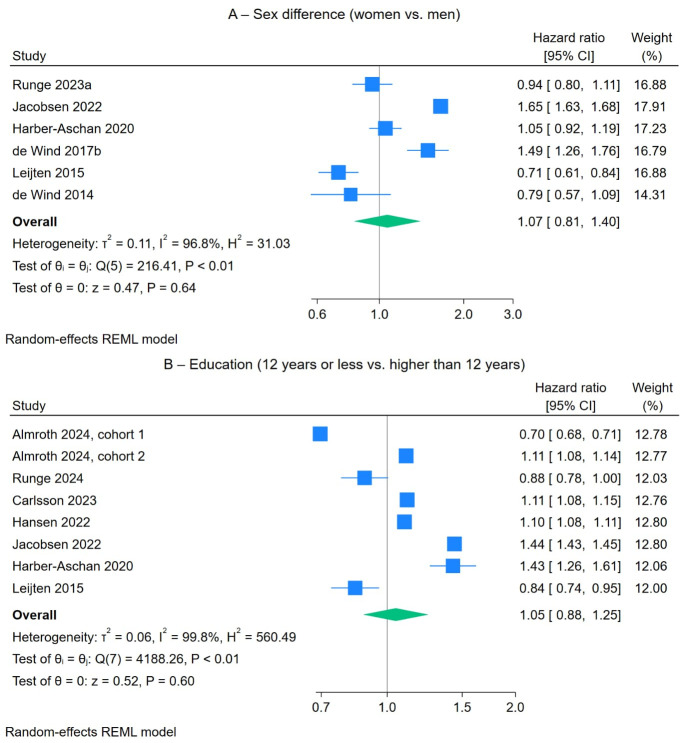
The effects of sex (A) and education (B) on voluntary early old-age retirement risk.

*Sociodemographic factors.* A 1-year increase in age was associated with voluntary early old-age retirement, with a HR of 1.35 (95% CI 1.12–1.63, table 2 and supplementary figure S3). The heterogeneity was high (99.7%) and was not accounted for by the methodological quality or sample size of the included studies. Additionally, excluding two studies (12, 71) that showed a small effect did not alter the heterogeneity. There was no significant difference in the risk of voluntary early old-age retirement between men and women (HR 1.07, 95% CI 0.81–1.40, six studies, figure 2A), between individuals with 12 years or less education and those with higher levels of education (HR 1.05, 95% CI 0.88–1.25, seven studies, figure 2B) nor between different occupational classes (supplementary figure S4). The risk was lower for single, divorced, or widowed workers compared to married or cohabiting workers (HR 0.74, 95% CI 0.60–0.91, four studies, table 2 and supplementary figure S5).

**Table 2:** Risk factors of voluntary early old-age retirement.

Risk factor			No. of studies	Sample	HR	95% CI	I^2^ statistic (%)
Sociodemographic factors							
	Age, 1-year increase		5	88 522	1.35	1.12–1.63	99.7
	Female sex		6	704 343	1.07	0.81–1.40	96.8
	Marital status (unmarried, separated, or widowed vs. married or cohabiting)		4	1 084 546	0.74	0.60–0.91	97.0
	Education, 12 years or less vs. higher level		7	2 193 988	1.05	0.88–1.25	99.8
Occupational class (ref: high)							
	Intermediate		3	66 599	0.95	0.69–1.30	91.1
	Low		3	66 599	0.85	0.39–1.82	97.4
Lifestyle factors							
	Smoking						
		Ever	2	10 461	1.01	0.97–1.06	0
		Past	2	10 461	1.00	0.93–1.08	0
		Current	2	10 461	1.02	0.97–1.08	0
	Body mass index						
		Overweight	2	10 461	1.11	1.04–1.19	0
		Obesity	2	10 461	1.05	0.92–1.19	0
		Overweight or obesity	2	10 461	1.10	1.03–1.17	0
Moderate or vigorous physical activity			2	10 461	1.13	1.04–1.22	0
Excessive alcohol intake			3	18 903	1.05	1.01–1.10	0
Occupational factors							
	Physically demanding work		6	141 545	1.22	1.00–1.49	95.7
	High emotional demands		3	10 390	1.01	0.93–1.09	0
	Low influence at work		2	7414	1.10	1.02–1.19	0
	Low job control		3	129 070	1.14	1.11–1.17	0
	Low job rewards		2	5288	1.06	0.95–1.18	0
	Low social support		2	8514	1.07	0.95–1.21	54.9
	Low predictability at work		2	2241	0.88	0.57–1.36	64.2
	Low quality of management		2	5495	1.10	0.97–1.26	48.4
	Low organizational justice		2	5130	1.27	1.10–1.46	0
	Low possibilities of skills and knowledge development		2	4193	2.16	1.63–2.85	0
Health conditions							
	Suboptimal self-rated general health (poor/moderate vs. good/excellent)		11	84 191	1.22	1-12–1.34	46.4
	Mental health conditions		11	222 231	1.16	0.99–1.37	73.2
		Depressive symptoms	6	15 314	1.34	1.12–1.61	39.6
	Chronic physical conditions		5	644 665	1.11	1.05–1.17	0
	Musculoskeletal disorders		2	64 176	1.10	0.98–1.22	0
	Cardiovascular disease		3	641 620	1.16	0.98–1.37	57.6
	Respiratory diseases		3	691 454	1.08	0.99–1.19	22.1
	Diabetes		3	641 620	1.02	0.96–1.09	15.1
	Daily activity limitations		5	16 338	1.16	0.99–1.36	0

*Lifestyle factors.* Overweight individuals (BMI 25.0–29.9 kg/m^2^) had an increased risk of voluntary early old-age retirement (HR 1.11, 95% CI 1.04–1.19, two studies, supplementary figure S6). The estimate for obesity (BMI > 30.0 kg/m^2^) did not reach statistical significance due to low statistical power. The HR for overweight or obesity (BMI > 25.0 kg/m^2^) was 1.10 (95% CI 1.03–1.17). Smoking was not related to voluntary early old-age retirement (supplementary figure S7), whereas moderate or high levels of alcohol consumption were associated with a small excess risk of voluntary early old-age retirement (HR 1.05, 95% CI 1.01–1.10, three studies, supplementary figure S8). Individuals with moderate or vigorous levels of physical activity during leisure time were more likely to retire early than those with low levels of leisure-time physical activity (HR 1.13, 95% CI 1.04–1.22, two studies, supplementary figure S9).

**Figure 3:**
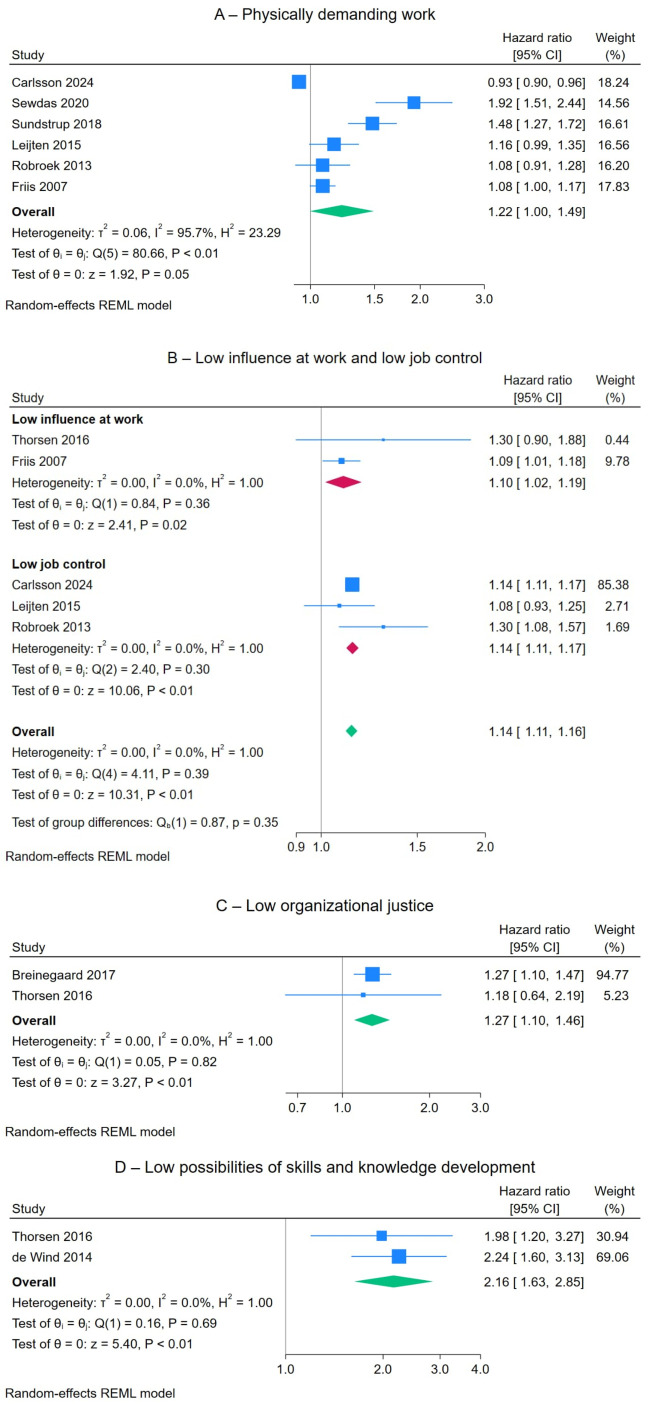
The effects of moderate or high physically demanding work (A), low influence at work and low job control (B), low organizational justice (C), and low possibilities of skills and knowledge development (D) on voluntary early old-age retirement risk.

*Occupational factors.* The following occupational factors were associated with an increased risk of voluntary early old-age retirement: physically demanding work (HR 1.29, 95% CI 1.05–1.59, five studies, figure 3A), low job control (HR 1.14, 95% CI 1.11–1.17, three studies, figure 3B), low influence at work (HR 1.10, 95% CI 1.02–1.19, two studies, figure 3B), low organizational justice (HR 1.27, 95% CI 1.10–1.46, two studies, figure 3C), and limited opportunities for skills and knowledge development (HR 2.16, 95% CI 1.63–2.85, two studies, figure 3D). High emotional demands, low job rewards, low social support, low predictability at work, and poor quality of management were not identified as factors contributing to voluntary early old-age retirement (supplementary figures S10–S14).

**Figure 4:**
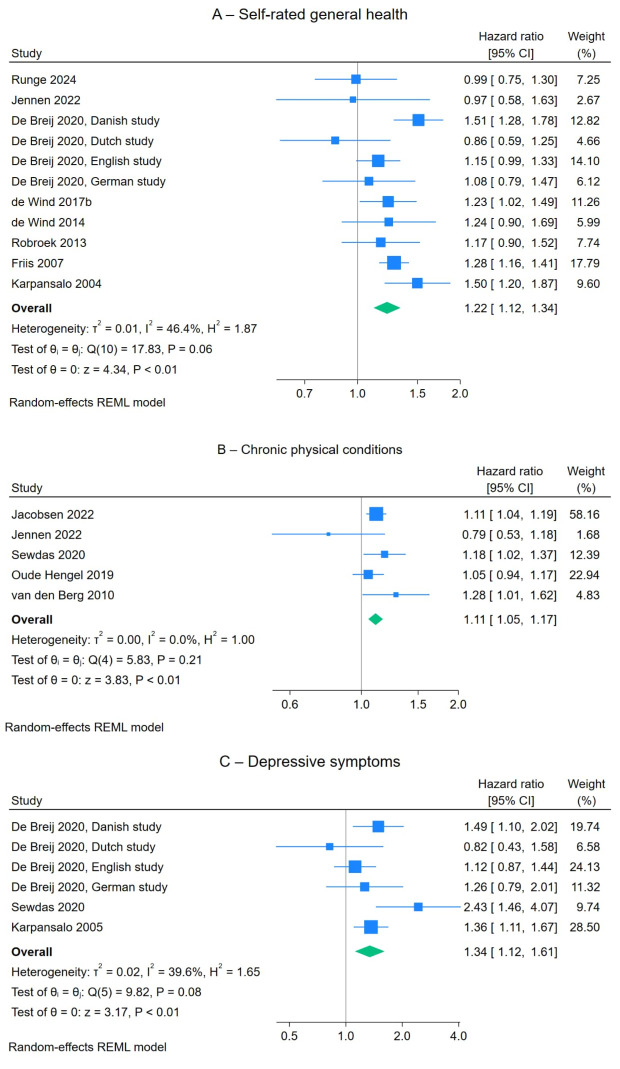
The effects of self-rated general health (A), chronic physical conditions (B), and depressive symptoms (C) on voluntary early old-age retirement.

*Health conditions.* Suboptimal self-rated general health (HR 1.22, 95% CI 1.12–1.34, eleven studies), chronic physical conditions (HR 1.11, 95% CI 1.05–1.17, five studies), and depressive symptoms (HR 1.34, 95% CI 1.12–1.61, six studies) were associated with increased risk of voluntary early old-age retirement (table 2 and figure 4). Mental health conditions (HR 1.16, 95% CI 0.99–1.37, eleven studies, P=0.07, supplementary figure S15), experiencing daily activity limitations (HR 1.16, 95% CI 0.99–1.36, P=0.07, five studies, supplementary figure S16), cardiovascular disease (HR 1.16, 95% CI 0.98–1.37, P=0.09, three studies, supplementary figure S17), and respiratory disease (HR 1.08, 95% CI 0.99–1.19, P=0.09, three studies, supplementary figure S18) were borderline statistically significantly associated with an increased risk of voluntary early old-age retirement. However, musculoskeletal disorders, and diabetes did not show a significant contribution to the risk (supplementary figures S19–S20). Meta-regression showed that the observed differences in effect size across various studies on self-rated general health, mental health conditions and depressive symptoms appeared to be independent of the methodological quality of the studies. However, for mental health conditions, smaller studies showed a larger effect size compared to larger studies. The pooled HR was 1.40 (95% CI 1.04–1.88, N=7840) for five smaller studies and 1.06 (95% CI 0.90–1.25, N=209 670) for six larger studies. There was no evidence of publication bias (supplementary figures S21–S22). In fact, using the trim-and-fill method suggested that four small studies showing a significant association were missing from the right side of the funnel plots for self-rated general health (supplementary figure S23).

## Discussion

The findings of this study indicate that for 40–64-year-old individuals, factors such as excess body mass, physically demanding jobs, psychosocial risk factors at work, lack of skills and knowledge development, suboptimal self-rated general health, and physical and mental health conditions are associated with an increased risk of voluntary early old-age retirement. These results suggest that to prolong the working careers of aging workers, it is essential to avoid overweight, ensure favorable physical and psychosocial working conditions, and maintain good health.

As expected, we found that older workers are more likely to retire early than younger workers. This trend is influenced by several factors: older workers often face health issues, experience layoffs, or feel dissatisfied with their work (73). Additionally, they may retire early to take care of family members or because they have accumulated sufficient savings (73). However, the rate of voluntary early old-age retirement did not differ between men and women, levels of education and occupational class. The included studies on sex, education and occupational class were highly heterogeneous and reported contradictory results. The lack of differences might be due to universal drivers for voluntary early old-age retirement, such as health issues, desire for leisure, and financial readiness, which can affect individuals regardless of their sex, education, or occupational class. Additionally, many retirement policies and incentives are designed to be broadly applicable, ensuring that all eligible employees, regardless of their background, have similar opportunities to retire early.

We observed that unpartnered (single, divorced, and/or widowed) older workers were less likely to voluntarily retire early compared to their partnered counterparts. A previous study suggested that the social significance of work partially mediated the link between marital status and early retirement by offering regular social interaction, which particularly benefited unpartnered older workers (74). Unpartnered older workers do not have a spouse at home who might prefer their partner to retire early (74). Conversely, workers with a partner who holds a positive attitude towards early retirement are more likely to retire early compared to those whose partners have a negative or neutral attitude or those without a partner (13). Furthermore, workers who have the financial means to stop working before reaching the official retirement age are more likely to opt for early retirement (13). This financial capability is often more common among partnered workers, as they may benefit from dual incomes and shared financial responsibilities, making early retirement a more feasible option.

Overweight and excessive alcohol consumption have been found to increase the risk of voluntary early old-age retirement. However, the pooled estimates were derived from only two studies on overweight/obesity and three studies on alcohol consumption, and the observed effects were relatively small. The two studies on obesity included in the analysis had low statistical power, which limits the reliability of their findings. Similarly, the overall meta-analysis also suffered from low statistical power, indicating a need for further research on this topic. The small effect of excessive alcohol consumption can be attributed to the comparison group used in the studies, which included abstainers, light drinkers, and moderate drinkers. It is important to note that both abstainers and moderate drinkers are at a higher risk of health conditions, as abstainers often stop drinking due to pre-existing health issues.

Workers who engaged in physical activities during their leisure time were more inclined to voluntarily retire early than those who were inactive. Individuals who prioritize leisure time physical activity might be more disciplined in financial planning and thus financially stable enough to retire early. They may value a balanced lifestyle and seek to retire early to spend more time on activities they enjoy. The observed link between physical activity and voluntary early old-age retirement might be coincidental, as only two studies have explored this connection. These studies categorized physical activity levels in a binary manner, defining “physically active” as engaging in exercise at least once a week or more. This limited scope suggests that further research is needed to confirm and better understand this potential relationship.

We observed higher rates of voluntary early old-age retirement among workers with low influence at work and limited opportunities for skills and knowledge development. This finding supports previous research indicating that a good psychosocial work environment promotes extended employment beyond retirement age (75). While high physical work demands were identified as a risk factor, a supportive psychosocial work environment appeared to facilitate extended employment even for those in physically demanding jobs (75). A poor psychosocial work environment and poor mental health may act as interconnected barriers to prolonged work careers. Low influence at work is linked to an increased risk of depressive symptoms (76), and poor self-rated general health (77). Additionally, limited opportunities for skills and knowledge development are associated with a decline in self-rated general health (77) and a higher likelihood of leaving current employment (25). These psychosocial factors may contribute to voluntary early old-age retirement through their impact on mental health conditions. The relationship between poor working conditions and voluntary early old-age retirement is complex and multifaceted. It is plausible that deteriorating mental health acts as a mediator in this association. A study found that effort–reward imbalance increased the risk of early labor market exit via a decrease in self-rated general health (78). Future research is needed to explore this potential mediation effect. Understanding this relationship could provide valuable insights for developing targeted interventions aimed at improving workplace conditions and supporting mental health, ultimately helping to reduce the incidence of voluntary early old-age retirement.

Physical (11) and mental (63, 70) health conditions are a major cause of labor force exit. Consistent with a previous meta-analysis of five studies (20), we identified an increased risk of voluntary early old-age retirement among middle-aged workers with poor self-rated health. The observed association between poor self-rated health and voluntary early old-age retirement are attributed to both physical and mental health conditions (79). We found that depressive symptoms are associated with a higher risk of voluntary early old-age retirement than physical conditions. In line with our results, a previous study (80) investigating factors associated with extending employment beyond retirement age found that the probability of extended employment was significantly higher in the absence of diagnosed mental disorders and among employees without symptoms of psychological distress, whereas the contribution of somatic conditions was modest. In our meta-analysis, chronic somatic conditions were modestly associated with an increased risk of voluntary early old-age retirement, a finding that aligns with a previous meta-analysis of five studies (20). This study suggests that addressing mental health conditions is crucial to mitigating their impact on voluntary early old-age retirement.

### Limitations of the included studies and the review

Each study included in this meta-analysis employed a longitudinal design and most studies recruited a sufficient number of participants, thereby ensuring adequate statistical power to examine risk factors for voluntary early old-age retirement. However, several limitations were identified within these studies. Notably, many studies did not account for a comprehensive range of potential confounding factors, with only 35% adjusting their observed associations for most of these factors. Such omissions could potentially exaggerate the reported associations. Moreover, 39% of the studies relied on self-reported data to assess voluntary early old-age retirement. One study included 16 psychosocial factors in a single multivariable model (64). Some of these factors may be highly correlated. After this overadjustment, the direction of association was even revered for poor leadership quality and poor predictability. However, these reverse associations were not statistically significant.

This review also encountered certain limitations. For some risk factors, the number of available studies was limited, resulting in low statistical power. Multiple meta-analyses, including those on leisure time physical activity, low influence at work, and low organizational justice, included only two studies. One of these studies was quite small, contributing minimally to the pooled risk estimates. Therefore, these risk estimates should be interpreted with caution. Additionally, a moderate to high level of heterogeneity was observed in certain meta-analyses. Consequently, further large-scale longitudinal studies are required to thoroughly investigate some risk factors. Our research was confined to studies published in English, which may introduce bias, as studies with non-significant findings are more likely to be published in local language journals. Despite this, our analysis encompassed a diverse array of risk factors for voluntary early old-age retirement. Most studies reviewed reported non-significant associations between some risk factors and voluntary early old-age retirement. In our literature search, we included three prominent American databases and the largest European database. Additionally, we used Google Scholar as a complementary resource. Given that PubMed, Web of Science, and Scopus are leading databases, the likelihood of missing significant studies is minimal. Lastly, the included studies were from European countries only. Therefore, the generalizability of our findings may be limited to regions with similar welfare regimes.

### Concluding remarks

The findings of this study highlight several key factors associated with an increased risk of voluntary early old-age retirement among middle-aged individuals. Specifically, excess body mass, physically demanding jobs, psychosocial risk factors at work, lack of skills and knowledge development, physical and mental health conditions significantly contribute to voluntary early old-age retirement. However, it is important to note that the number of studies examining some of these risk factors was limited, which may affect the robustness of these findings. To prolong working careers of aging workers, targeted interventions and programs are necessary to address these factors by encouraging healthy lifestyle choices, fostering a supportive work environment, providing opportunities for skill development, improving organizational justice, increasing job influence and control, and promoting health programs and resources to manage health conditions.

## Supplementary material

Supplementary material

## References

[1] Kadefors R, Nilsson K, Östergren PO, Rylander L, Albin M. Social inequality in working life expectancy in Sweden. Z Gerontol Geriatr 2019 Feb;52 Suppl 1:52–61. 10.1007/s00391-018-01474-3 30413944 PMC6373384

[2] OECD. Flexible retirement in OECD countries, in Pensions at a Glance 2017: OECD and G20 Indicators, OECD Publishing, Paris, 10.1787/pension_glance-2017-5-en. 2017.

[3] Robroek SJ, Schuring M, Croezen S, Stattin M, Burdorf A. Poor health, unhealthy behaviors, and unfavorable work characteristics influence pathways of exit from paid employment among older workers in Europe: a four year follow-up study. Scand J Work Environ Health 2013 Mar;39(2):125–33. 10.5271/sjweh.3319 22949091

[4] Leijten FR, de Wind A, van den Heuvel SG, Ybema JF, van der Beek AJ, Robroek SJ et al. The influence of chronic health problems and work-related factors on loss of paid employment among older workers. J Epidemiol Community Health 2015 Nov;69(11):1058–65. 10.1136/jech-2015-205719 26112957

[5] Reeuwijk KG, van Klaveren D, van Rijn RM, Burdorf A, Robroek SJ. The influence of poor health on competing exit routes from paid employment among older workers in 11 European countries. Scand J Work Environ Health 2017 Jan;43(1):24–33. 10.5271/sjweh.3601 27829251

[6] Risk Factors Collaborators GB; GBD 2021 Risk Factors Collaborators. Global burden and strength of evidence for 88 risk factors in 204 countries and 811 subnational locations, 1990-2021: a systematic analysis for the Global Burden of Disease Study 2021. Lancet 2024 May;403(10440):2162–203. 10.1016/S0140-6736(24)00933-4 38762324 PMC11120204

[7] GBD 2021 Diseases and Injuries Collaborators. Global incidence, prevalence, years lived with disability (YLDs), disability-adjusted life-years (DALYs), and healthy life expectancy (HALE) for 371 diseases and injuries in 204 countries and territories and 811 subnational locations, 1990-2021: a systematic analysis for the Global Burden of Disease Study 2021. Lancet 2024 May;403(10440):2133–61. 10.1016/S0140-6736(24)00757-8 38642570 PMC11122111

[8] Runge K, van Zon SK, Henkens K, Bültmann U. Metabolic syndrome and poor self-rated health as risk factors for premature employment exit: a longitudinal study among 55 016 middle-aged and older workers from the Lifelines Cohort Study and Biobank. Eur J Public Health 2024 Apr;34(2):309–15. 10.1093/eurpub/ckad219 38110727 PMC10990532

[9] Breinegaard N, Jensen JH, Bonde JP. Organizational change, psychosocial work environment, and non-disability early retirement: a prospective study among senior public employees. Scand J Work Environ Health 2017 May;43(3):234–40. 10.5271/sjweh.3624 28166362

[10] Friis K, Ekholm O, Hundrup YA, Obel EB, Grønbaek M. Influence of health, lifestyle, working conditions, and sociodemography on early retirement among nurses: the Danish Nurse Cohort Study. Scand J Public Health 2007;35(1):23–30. 10.1080/14034940600777278 17366084

[11] Jacobsen PA, Kragholm K, Andersen MP, Lindgren FL, Ringgren KB, Torp-Pedersen C et al. Voluntary early retirement and mortality in patients with and without chronic diseases: a nationwide Danish Registry study. Public Health 2022 Oct;211:114–21. 10.1016/j.puhe.2022.07.019 36088807

[12] Wind A, Burr H, Pohrt A, Hasselhorn HM, Van der Beek AJ, Rugulies R. The association of health and voluntary early retirement pension and the modifying effect of quality of supervision: results from a Danish register-based follow-up study. Scand J Public Health 2017 b Jul;45(5):468–75. 10.1177/1403494817699998 28381121 PMC5495428

[13] de Wind A, Geuskens GA, Ybema JF, Blatter BM, Burdorf A, Bongers PM et al. Health, job characteristics, skills, and social and financial factors in relation to early retirement--results from a longitudinal study in the Netherlands. Scand J Work Environ Health 2014 Mar;40(2):186–94. 10.5271/sjweh.3393 24132500

[14] Page MJ, McKenzie JE, Bossuyt PM, Boutron I, Hoffmann TC, Mulrow CD et al. The PRISMA 2020 statement: an updated guideline for reporting systematic reviews. BMJ 2021 Mar;372:n71. 10.1136/bmj.n71 33782057 PMC8005924

[15] Shiri R, Poutanen J, Härmä M, Ervasti J, Haukka E. A meta-analysis of unemployment risk factors for middle-aged workers. Scand J Work Environ Health 2025 May;51(3):135–45. 10.5271/sjweh.4216 40036720 PMC12068247

[16] Knardahl S, Johannessen HA, Sterud T, Härmä M, Rugulies R, Seitsamo J et al. The contribution from psychological, social, and organizational work factors to risk of disability retirement: a systematic review with meta-analyses. BMC Public Health 2017 Feb;17(1):176. 10.1186/s12889-017-4059-4 28178966 PMC5299735

[17] Amiri S, Behnezhad S. Smoking and disability pension: a systematic review and meta-analysis. Public Health 2020 Sep;186:297–303. 10.1016/j.puhe.2020.04.013 32882482

[18] Shiri R, Falah-Hassani K, Lallukka T. Body mass index and the risk of disability retirement: a systematic review and meta-analysis. Occup Environ Med 2020 Jan;77(1):48–55. 10.1136/oemed-2019-105876 31467042

[19] Robroek SJ, Reeuwijk KG, Hillier FC, Bambra CL, van Rijn RM, Burdorf A. The contribution of overweight, obesity, and lack of physical activity to exit from paid employment: a meta-analysis. Scand J Work Environ Health 2013 May;39(3):233–40. 10.5271/sjweh.3354 23460255

[20] van Rijn RM, Robroek SJ, Brouwer S, Burdorf A. Influence of poor health on exit from paid employment: a systematic review. Occup Environ Med 2014 Apr;71(4):295–301. 10.1136/oemed-2013-101591 24169931

[21] Amiri S, Behnezhad S. Depression and risk of disability pension: A systematic review and meta-analysis. Int J Psychiatry Med 2019 May;91217419837412:91217419837412. 10.1177/0091217419837412 31060410

[22] Neovius K, Johansson K, Rössner S, Neovius M. Disability pension, employment and obesity status: a systematic review. Obes Rev 2008 Nov;9(6):572–81. 10.1111/j.1467-789X.2008.00502.x 18518906

[23] Carlsson E, Hemmingsson T, Landberg J, Burström B, Thern E. Do early life factors explain the educational differences in early labour market exit? A register-based cohort study. BMC Public Health 2023 Aug;23(1):1680. 10.1186/s12889-023-16626-3 37653490 PMC10472566

[24] de Wind A, Geuskens GA, Ybema JF, Bongers PM, van der Beek AJ. The role of ability, motivation, and opportunity to work in the transition from work to early retirement--testing and optimizing the Early Retirement Model. Scand J Work Environ Health 2015 Jan;41(1):24–35. 10.5271/sjweh.3468 25393088

[25] Shiri R, El-Metwally A, Sallinen M, Pöyry M, Härmä M, Toppinen-Tanner S. The Role of Continuing Professional Training or Development in Maintaining Current Employment: A Systematic Review. Healthcare (Basel) 2023 Nov;11(21):2900. 10.3390/healthcare11212900 37958044 PMC10647344

[26] Karpansalo M, Manninen P, Kauhanen J, Lakka TA, Salonen JT. Perceived health as a predictor of early retirement. Scand J Work Environ Health 2004 Aug;30(4):287–92. 10.5271/sjweh.796 15458011

[27] Almroth M, Falkstedt D, Hemmingsson T, Albin M, Badarin K, Selander J et al. Labour market exit routes in high- and low-educated older workers before and after social insurance and retirement policy reforms in Sweden. 2025;45(6):1228–47. 10.1017/S0144686X24000047.

[28] Carlsson E, Hemmingsson T, Almroth M, Falkstedt D, Kjellberg K, Thern E. Mediating effect of working conditions on the association between education and early labour market exit: a cohort study of Swedish men. Occup Environ Med 2024 Dec;81(11):547–55. 10.1136/oemed-2024-109594 39586667 PMC11671893

[29] Carlsson E, Hemmingsson T, Landberg J, Burström B, Thern E. The contribution of common mental disorders and alcohol-related morbidity to educational differences in early labour market exit among older workers: a register-based cohort study. Eur J Public Health 2025 Feb;35(1):65–71. 10.1093/eurpub/ckae212 39798163 PMC11832143

[30] Morois S, Lemogne C, Leclerc A, Limosin F, Goldberg S, Goldberg M et al. More than Light Alcohol Consumption Predicts Early Cessation from Employment in French Middle-Aged Men. Alcohol Alcohol 2016 Mar;51(2):224–31. 10.1093/alcalc/agv092 26271114

[31] Sundstrup E, Hansen AM, Mortensen EL, Poulsen OM, Clausen T, Rugulies R et al. Retrospectively assessed physical work environment during working life and risk of sickness absence and labour market exit among older workers. Occup Environ Med 2018 Feb;75(2):114–23. 10.1136/oemed-2016-104279 28819019 PMC5800344

[32] Hansen TH, Vignes B. Early retirement from the labour market among immigrants and natives: A register-based study of Norway. Nordic Welfare Research. 2022;7(2):75–95. 10.18261/nwr.7.2.1

[33] Harber-Aschan L, Chen WH, McAllister A, Koitzsch Jensen N, Thielen K, Andersen I et al. The impact of longstanding illness and common mental disorder on competing employment exits routes in older working age: A longitudinal data-linkage study in Sweden. PLoS One 2020 Feb;15(2):e0229221. 10.1371/journal.pone.0229221 32097437 PMC7041791

[34] Higgins JP, Green S, editors. Cochrane Handbook for Systematic Reviews of Interventions Version 5.1.0 [updated March 2011]. The Cochrane Collaboration, 2011. Available from: www.handbook.cochrane.org

[35] Rice NE, Lang IA, Henley W, Melzer D. Common health predictors of early retirement: findings from the English Longitudinal Study of Ageing. Age Ageing 2011 Jan;40(1):54–61. 10.1093/ageing/afq153 21148324

[36] Gong CH, He X. Factors Predicting Voluntary and Involuntary Workforce Transitions at Mature Ages: evidence from HILDA in Australia. Int J Environ Res Public Health 2019 Oct;16(19):3769. 10.3390/ijerph16193769 31597239 PMC6801955

[37] Dong L, Agnew J, Mojtabai R, Surkan PJ, Spira AP. Insomnia as a predictor of job exit among middle-aged and older adults: results from the Health and Retirement Study. J Epidemiol Community Health 2017 Aug;71(8):750–7. 10.1136/jech-2016-208630 28298414

[38] Olesen SC, Butterworth P, Rodgers B. Is poor mental health a risk factor for retirement? Findings from a longitudinal population survey. Soc Psychiatry Psychiatr Epidemiol 2012 May;47(5):735–44. 10.1007/s00127-011-0375-7 21461932

[39] Sundstrup E, Thorsen SV, Rugulies R, Larsen M, Thomassen K, Andersen LL. Importance of the Working Environment for Early Retirement: Prospective Cohort Study with Register Follow-Up. Int J Environ Res Public Health 2021 Sep;18(18):9817. 10.3390/ijerph18189817 34574740 PMC8472036

[40] Takada M, Tabuchi T, Iso H. Newly diagnosed disease and job loss: a nationwide longitudinal study among middle-aged Japanese. Occup Environ Med 2021 Apr;78(4):279–85. 10.1136/oemed-2020-106685 33268468

[41] Mein G, Martikainen P, Stansfeld SA, Brunner EJ, Fuhrer R, Marmot MG. Predictors of early retirement in British civil servants. Age Ageing 2000 Nov;29(6):529–36. 10.1093/ageing/29.6.529 11191246

[42] Pan T, Mercer SW, Zhao Y, McPake B, Desloge A, Atun R et al. The association between mental-physical multimorbidity and disability, work productivity, and social participation in China: a panel data analysis. BMC Public Health 2021 Feb;21(1):376. 10.1186/s12889-021-10414-7 33602174 PMC7890601

[43] Mäcken J. Work stress among older employees in Germany: effects on health and retirement age. PLoS One 2019 Feb;14(2):e0211487. 10.1371/journal.pone.0211487 30716089 PMC6361437

[44] Schinkel-Ivy A, Mosca I, Mansfield A. Factors Contributing to Unexpected Retirement and Unemployment in Adults Over 50 Years Old in Ireland. Gerontol Geriatr Med 2017 Jul;3:2333721417722709. 10.1177/2333721417722709 28808669 PMC5536377

[45] Chen WH. Health and transitions into nonemployment and early retirement among older workers in Canada. Econ Hum Biol 2019 Dec;35:193–206. 10.1016/j.ehb.2019.06.001 31446313

[46] Houston DK, Cai J, Stevens J. Overweight and obesity in young and middle age and early retirement: the ARIC study. Obesity (Silver Spring) 2009 Jan;17(1):143–9. 10.1038/oby.2008.464 19107127 PMC5774854

[47] Whittaker W, Higgerson J, Eden M, Payne K, Wilkie R, Verstappen SM. Effects of employees living with an ‘arthritis’ on sickness absence and transitions out of employment: a comparative observational study in the UK. RMD Open 2024 Nov;10(4):e004817. 10.1136/rmdopen-2024-004817 39615887 PMC11624701

[48] Stynen D, Jansen NW, Kant I. The impact of work-related and personal resources on older workers’ fatigue, work enjoyment and retirement intentions over time. Ergonomics 2017 Dec;60(12):1692–707. 10.1080/00140139.2017.1334094 28532293

[49] Nexo MA, Borg V, Sejbaek CS, Carneiro IG, Hjarsbech PU, Rugulies R. Depressive symptoms and early retirement intentions among Danish eldercare workers: cross-sectional and longitudinal analyses. BMC Public Health 2015 Jul;15:677. 10.1186/s12889-015-1973-1 26184519 PMC4504417

[50] von Bonsdorff ME, Huuhtanen P, Tuomi K, Seitsamo J. Predictors of employees’ early retirement intentions: an 11-year longitudinal study. Occup Med (Lond) 2010 Mar;60(2):94–100. 10.1093/occmed/kqp126 19734239

[51] Muurinen C, Laine M, Pentti J, Virtanen M, Salo P, Kivimäki M et al. Vertical and horizontal trust at work as predictors of retirement intentions: the Finnish Public Sector Study. PLoS One 2014 Sep;9(9):e106956. 10.1371/journal.pone.0106956 25191745 PMC4156392

[52] Sejbaek CS, Nexo MA, Borg V. Work-related factors and early retirement intention: a study of the Danish eldercare sector. Eur J Public Health 2013 Aug;23(4):611–6. 10.1093/eurpub/cks117 22930740

[53] Bethge M, Radoschewski FM, Gutenbrunner C. The Work Ability Index as a screening tool to identify the need for rehabilitation: longitudinal findings from the Second German Sociomedical Panel of Employees. J Rehabil Med 2012 Nov;44(11):980–7. 10.2340/16501977-1063 23027375

[54] Szubert Z, Sobala W. Current determinants of early retirement among blue collar workers in Poland. Int J Occup Med Environ Health 2005;18(2):177–84. 16201209

[55] Pan T, Mercer SW, Zhao Y, McPake B, Desloge A, Atun R et al. The association between mental-physical multimorbidity and disability, work productivity, and social participation in China: a panel data analysis. BMC Public Health 2021 Feb;21(1):376. 10.1186/s12889-021-10414-7 33602174 PMC7890601

[56] Park J. Health factors and early retirement among older workers. Perspect Labour Income 2010;11(6):5–13.

[57] Van Solinge H, Henkens K. Work-related factors as predictors in the retirement decision-making process of older workers in the Netherlands. Ageing Soc 2014;34(9):1551–74. 10.1017/S0144686X13000330

[58] Hale L, Singer L, Barnet JH, Peppard PE, Hagen EW. Associations Between Midlife Insomnia Symptoms and Earlier Retirement. Sleep Health 2017 Jun;3(3):170–7. 10.1016/j.sleh.2017.03.003 28526254 PMC7921848

[59] Riekhoff AJ, Järnefelt N, Laaksonen M. Workforce Composition and the Risk of Labor Market Exit Among Older Workers in Finnish Companies. Work Aging Retire 2020;6(2):88–100. 10.1093/workar/waz023

[60] Runge K, van Zon SK, Henkens K, Bültmann U. Metabolic syndrome increases the risk for premature employment exit: A longitudinal study among 60 427 middle-aged and older workers from the Lifelines Cohort Study and Biobank. Scand J Work Environ Health 2023 Nov;49(8):569–77. 10.5271/sjweh.4113 37672668 PMC10866619

[61] Jennen JG, Jansen NW, van Amelsvoort LG, Slangen JJ, Kant I. Chronic conditions and self-perceived health among older employees in relation to indicators of labour participation and retirement over time. Work 2022;71(1):133–50. 10.3233/WOR-210436 34924423 PMC8842761

[62] De Breij S, Mäcken J, Qvist JY, Holman D, Hess M, Huisman M et al. Educational differences in the influence of health on early work exit among older workers. Occup Environ Med 2020 Aug;77(8):568–75. 10.1136/oemed-2019-106253 32269132 PMC7402445

[63] Sewdas R, Thorsen SV, Boot CR, Bjørner JB, Van der Beek AJ. Determinants of voluntary early retirement for older workers with and without chronic diseases: A Danish prospective study. Scand J Public Health 2020 Mar;48(2):190–9. 10.1177/1403494819852787 31319774 PMC7042495

[64] Thorsen SV, Jensen PH, Bjørner JB. Psychosocial work environment and retirement age: a prospective study of 1876 senior employees. Int Arch Occup Environ Health 2016 Aug;89(6):891–900. 10.1007/s00420-016-1125-7 27055542

[65] Oude Hengel K, Robroek SJ, Eekhout I, van der Beek AJ, Burdorf A. Educational inequalities in the impact of chronic diseases on exit from paid employment among older workers: a 7-year prospective study in the Netherlands. Occup Environ Med 2019 Oct;76(10):718–25. 10.1136/oemed-2019-105788 31409626 PMC6817992

[66] Lund T, Villadsen E. Who retires early and why? Determinants of early retirement pension among Danish employees 57-62 years. Eur J Ageing 2005 Nov;2(4):275–80. 10.1007/s10433-005-0013-x 28794742 PMC5546287

[67] de Wind A, Leijten FR, Hoekstra T, Geuskens GA, Burdorf A, van der Beek AJ. “Mental retirement?” Trajectories of work engagement preceding retirement among older workers. Scand J Work Environ Health 2017 a Jan;43(1):34–41. 10.5271/sjweh.3604 27907223

[68] Kouwenhoven-Pasmooij TA, Burdorf A, Roos-Hesselink JW, Hunink MG, Robroek SJ. Cardiovascular disease, diabetes and early exit from paid employment in Europe; the impact of work-related factors. Int J Cardiol 2016 Jul;215:332–7. 10.1016/j.ijcard.2016.04.090 27128556

[69] van den Berg T, Schuring M, Avendano M, Mackenbach J, Burdorf A. The impact of ill health on exit from paid employment in Europe among older workers. Occup Environ Med 2010 Dec;67(12):845–52. 10.1136/oem.2009.051730 20798020

[70] Karpansalo M, Kauhanen J, Lakka TA, Manninen P, Kaplan GA, Salonen JT. Depression and early retirement: prospective population based study in middle aged men. J Epidemiol Community Health 2005 Jan;59(1):70–4. 10.1136/jech.2003.010702 15598730 PMC1763370

[71] Blekesaune M, Solem PE. Working conditions and early retirement - A prospective study of retirement behavior. Res Aging 2005;27(1):3–30. 10.1177/0164027504271438

[72] Stynen D, Jansen NW, Slangen JJ, de Grip A, Kant IJ. Need for recovery and different types of early labour force exit: a prospective cohort study among older workers. Int Arch Occup Environ Health 2019 Jul;92(5):683–97. 10.1007/s00420-019-01404-9 30746558 PMC6556172

[73] Reeuwijk KG, de Wind A, Westerman MJ, Ybema JF, van der Beek AJ, Geuskens GA. ‘All those things together made me retire’: qualitative study on early retirement among Dutch employees. BMC Public Health 2013 May;13:516. 10.1186/1471-2458-13-516 23714371 PMC3674915

[74] Eismann M, Henkens K, Kalmijn M. Why Singles Prefer to Retire Later. Res Aging 2019 Dec;41(10):936–60. 10.1177/0164027519873537 31500549 PMC6838727

[75] Andersen LL, Thorsen SV, Larsen M, Sundstrup E, Boot CR, Rugulies R. Work factors facilitating working beyond state pension age: prospective cohort study with register follow-up. Scand J Work Environ Health 2021 Jan;47(1):15–21. 10.5271/sjweh.3904 32463101 PMC7801141

[76] Burr H, Rauch A, Rose U, Tisch A, Tophoven S. Employment status, working conditions and depressive symptoms among German employees born in 1959 and 1965. Int Arch Occup Environ Health 2015 Aug;88(6):731–41. 10.1007/s00420-014-0999-5 25416510 PMC4508361

[77] Burr H, Hasselhorn HM, Kersten N, Pohrt A, Rugulies R. Does age modify the association between psychosocial factors at work and deterioration of self-rated health? Scand J Work Environ Health 2017 Sep;43(5):465–74. 10.5271/sjweh.3648 28553992

[78] Toczek L, Peter R. Investigating the influence of work-related stress on early labour market exit: the role of health. Eur J Ageing 2023 Jul;20(1):31. 10.1007/s10433-023-00778-7 37405533 PMC10323059

[79] Levinson D, Kaplan G. What does Self Rated Mental Health Represent. J Public Health Res 2014 Dec;3(3):287. 10.4081/jphr.2014.287 25553310 PMC4274494

[80] Virtanen M, Oksanen T, Batty GD, Ala-Mursula L, Salo P, Elovainio M et al. Extending employment beyond the pensionable age: a cohort study of the influence of chronic diseases, health risk factors, and working conditions. PLoS One 2014 Feb;9(2):e88695. 10.1371/journal.pone.0088695 24586372 PMC3929527

