# Supplementary material for: Risk factors for voluntary early old-age retirement in middle-aged workers: A meta-analysis
Source: Scand J Work Environ Health. 2025 Oct 30;51(6):458–71. doi: 10.5271/sjweh.4241 (PMC12588187; doi:10.5271/sjweh.4241)
Supplement: Supplementary material [file SJWEH-51-458-S001.pdf]

Supplementary figures and tables

Risk factors for voluntary early old-age retirement in middle-aged workers: A meta-analysis

Rahman Shiri, PhD,[1] Joonas Poutanen, MSc,[1] Eija Haukka, PhD,[1] Mikko Härmä, PhD,[1] Jenni Ervasti, PhD [1]

[1] Finnish Institute of Occupational Health, Helsinki, Finland.

No. of studies	First author, year (reference no.)	Selection	Performance	Detection	Confounding	Attrition
1	Carlsson 2025 (29)	Low	Low	Low	High	Low
1	Carlsson 2024 (28)	Low	Moderate	Low	High	Low
1	Carlsson 2023 (23)	Low	Low	Low	Moderate	Low
2	Almroth 2024 (27)	Low	Low	Low	Moderate	Low
3	Runge 2024 (8) & Runge 2023 (60)	Moderate	Moderate	Moderate	Low	Moderate
4	Hansen 2022 (32)	Low	Low	Low	High	Low
5	Jacobsen 2022 (11)	Low	Low	Low	Moderate	Low
6	Jennen 2022 (61)	High	Moderate	Moderate	Low	Low
7	De Breij 2020 (62), the Dutch study	Moderate	Moderate	Moderate	Moderate	Moderate
8	De Breij 2020 (62), the Danish study	Moderate	Moderate	Moderate	Moderate	Moderate
9	De Breij 2020 (62), the English study	Moderate	Moderate	Moderate	Moderate	Moderate
10	De Breij 2020 (62), the German study	High	Moderate	Moderate	Moderate	High
11	Harber–Aschan 2020 (33)	Moderate	Moderate	Low	Low	Moderate
12	Sewdas 2020 (63)	Moderate	Moderate	Low	Moderate	Low
12	Thorsen 2016 (64)	Moderate	Moderate	Low	Low	Low
13	Oude Hengel 2019 (65)	Moderate	Moderate	Low	Moderate	Low
14	Stynen 2019 (72)	High	Moderate	Moderate	Moderate	Low
15	Sundstrup 2018 (31)	High	Moderate	Low	Low	Low
16	Breinegaard 2017 (9)	Moderate	Moderate	Low	Moderate	Low
17	de Wind 2017b (12) & Lund 2005 (66)	Moderate	Moderate	Low	Moderate	Low
18	de Wind 2017a (67), de Wind 2015 (24) & Leijten 2015 (4)	Moderate	Moderate	Moderate	Moderate	Moderate
18	de Wind 2014 (13)	Moderate	Moderate	Moderate	Moderate	Low
19	Reeuwijk 2017 (5), Kouwenhoven–Pasmooij 2016 (68) & van den Berg 2010 (69)	Moderate	Moderate	Moderate	Moderate	Moderate
19	Robroek 2013 (3)	Moderate	Moderate	Moderate	Low	Moderate
20	Morois 2016 (30)	High	Moderate	Low	Moderate	Low
21	Friis 2007 (10)	Low	Moderate	Low	Low	Low
22	Blekesaune 2005 (71)	Moderate	Moderate	Low	Moderate	Low
23	Karpansalo 2005 (70) & Karpansalo 2004 (26)	Low	Moderate	Low	Low	Low

Low risk (green)
Moderate risk (yellow)
High risk (red)

Supplementary Figure S1: The quality assessment (risk of bias)

No. of studies	First author, year (reference no.)	Overall	BMI	Smoking, alcohol consumption, or physical activity	Occupational factors	Work ability, perceived general health, or sleep	Metabolic syndrome, or liver function tests	Medical conditions, use of medicines, or physical capability
1	Carlsson 2025 (29)	🟢						🟢
1	Carlsson 2024 (28)	🟡			🟡			
1	Carlsson 2023 (23)	🟢						
2	Almroth 2024 (27)	🟢						
3	Runge 2024 (8) & Runge 2023 (60)	🟡			🟡	🟡	🟢	🟡
4	Hansen 2022 (32)	🟢						
5	Jacobsen 2022 (11)	🟢						🟢
6	Jennen 2022 (61)	🟡				🟡		🟡
7	De Breij 2020 (62), the Dutch study	🟡				🟡		🟡
8	De Breij 2020 (62), the Danish study	🟡				🟡		🔴
9	De Breij 2020 (62), the English study	🟡				🟡		🟡
10	De Breij 2020 (62), the German study	🟡				🟡		🟡
11	Harber–Aschan 2020 (33)	🟡						🟡
12	Sewdas 2020 (63)	🟡			🟡	🟡		🟡
12	Thorsen 2016 (64)	🟡			🟡			
13	Oude Hengel 2019 (65)	🟡						🟡
14	Stynen 2019 (72)	🟡			🟡			
15	Sundstrup 2018 (31)	🟡			🟡			
16	Breinegaard 2017 (9)	🟡			🟡			
17	de Wind 2017b (12) & Lund 2005 (66)	🟡			🟡	🟡		
18	de Wind 2017a (67), de Wind 2015 (24) & Leijten 2015 (4)	🟡			🟡	🟡		🟡
18	de Wind 2014 (13)	🟡			🟡	🟡		
19	Reeuwijk 2017 (5) , Kouwenhoven–Pasmooij 2016 (68) & van den Berg 2010 (69)	🟡				🟡		🟡
19	Robroek 2013 (3)	🟡	🟡	🟡	🟡			🟡
20	Morois 2016 (30)	🟡		🟡				
21	Friis 2007 (10)	🟡	🟡	🟡	🟡	🟡		
22	Blekesaune 2005 (71)	🟡			🟡			
23	Karpansalo 2005 (70) & Karpansalo 2004 (26)	🟡				🟡		🟡

Low risk 🟢
Moderate risk 🟡
High risk 🔴

Supplementary Figure S2: The risk of performance bias.

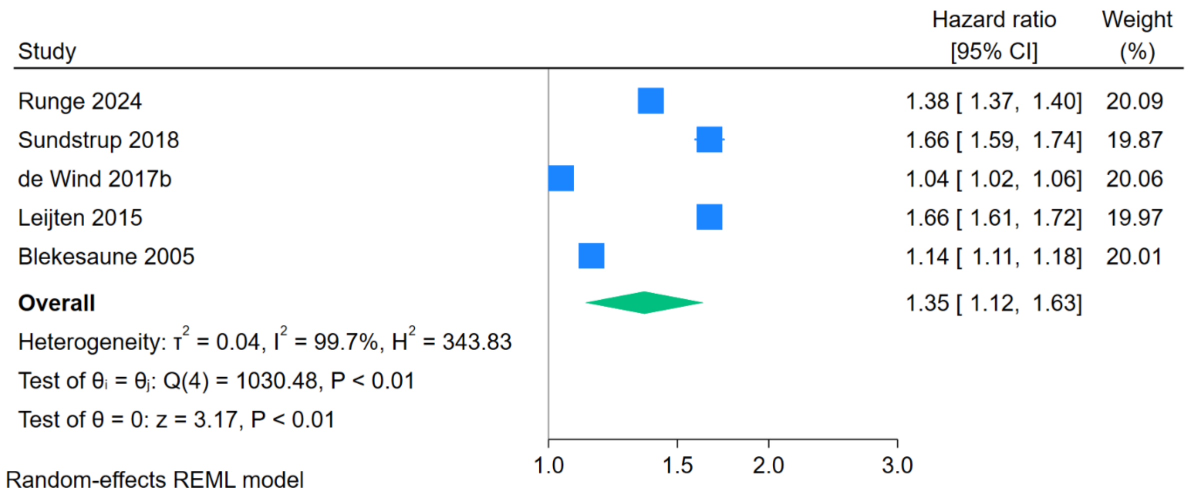

Figure S3: The effects of 1-year increase in age on voluntary early old-age retirement.

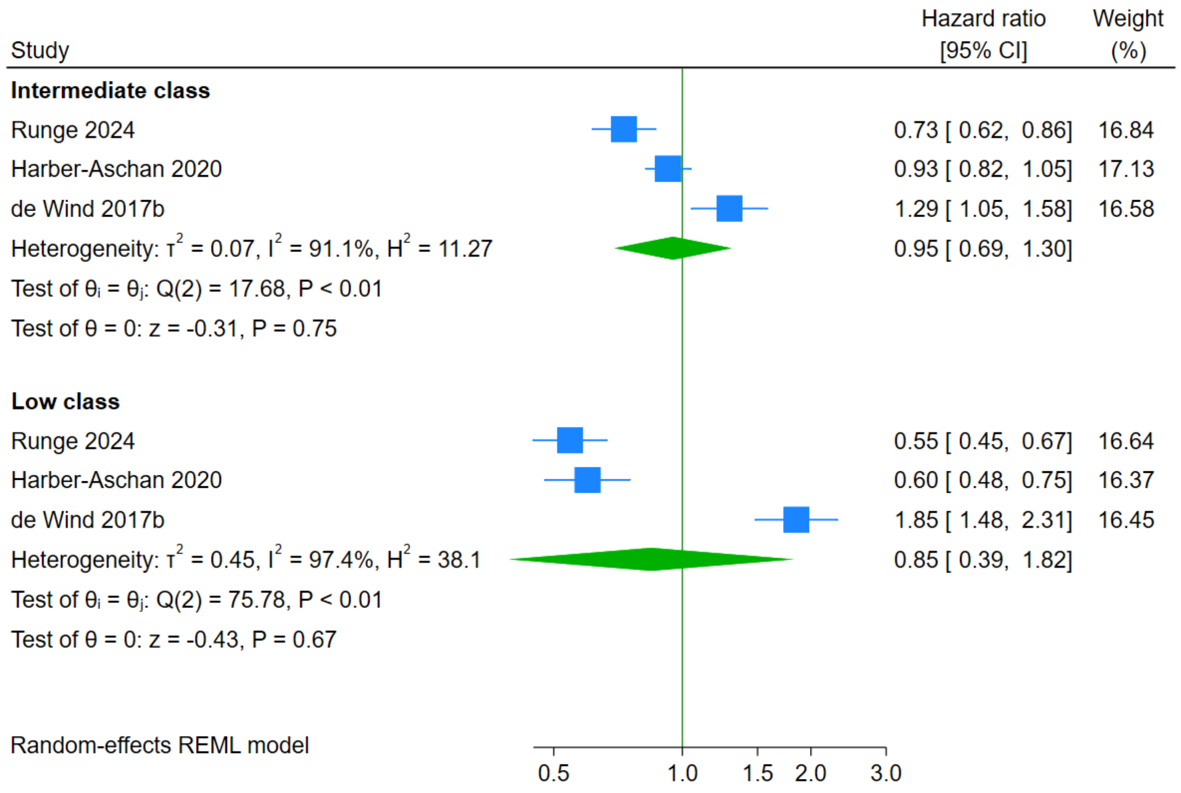

Figure S4: The effect of occupational class on voluntary early old-age retirement (intermediate or low vs. high).

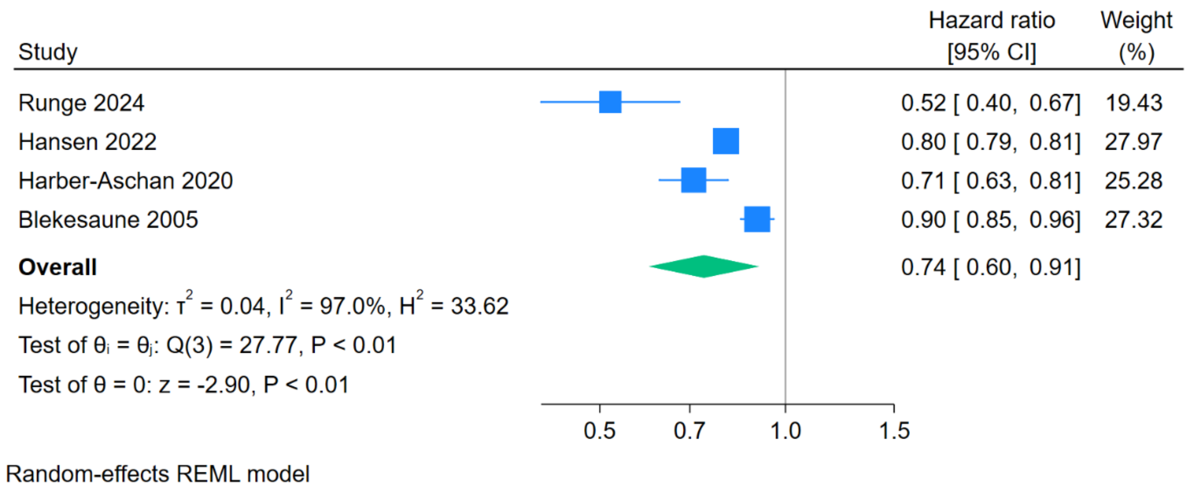

Figure S5: The effect of marital status on voluntary early old-age retirement (single, divorced or widowed vs. married or cohabiting).

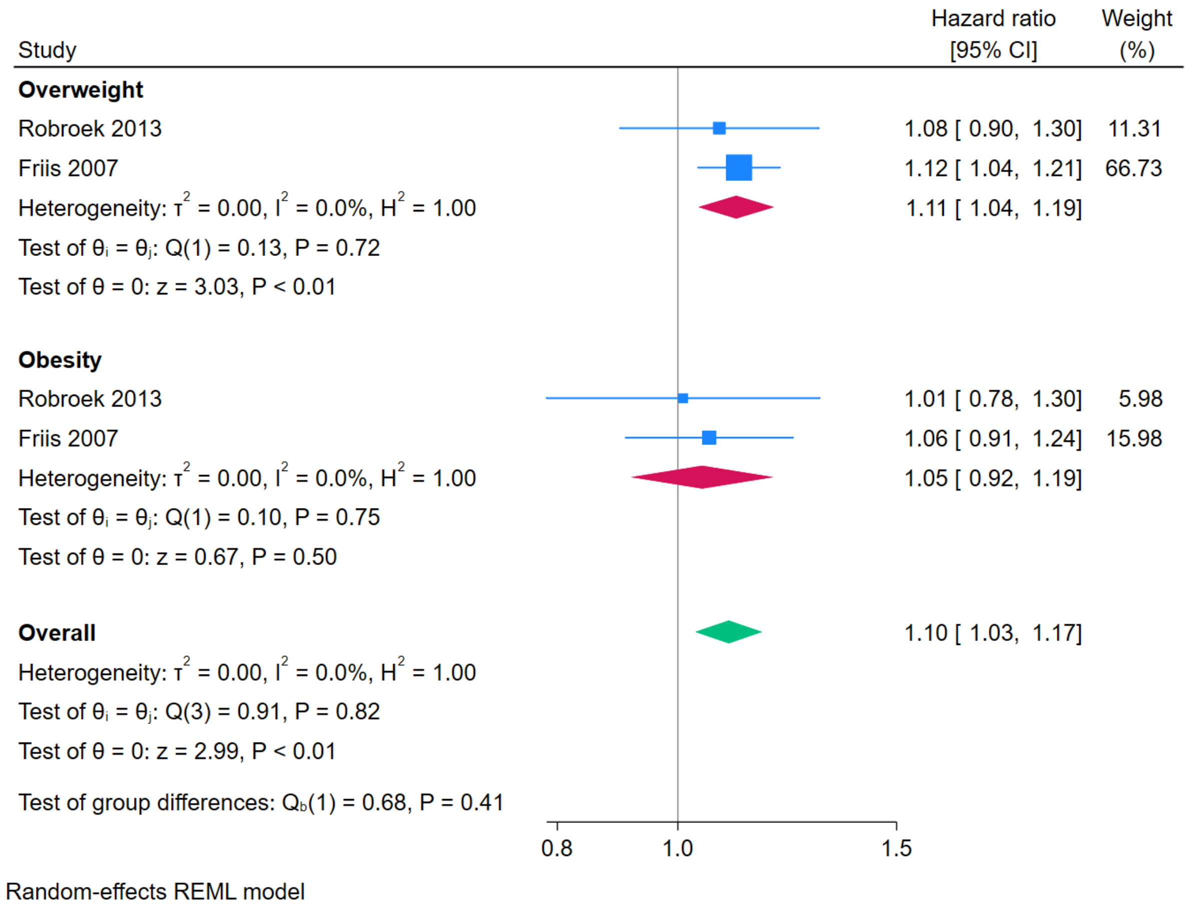

Figure S6: The effect of body mass index on voluntary early old-age retirement.

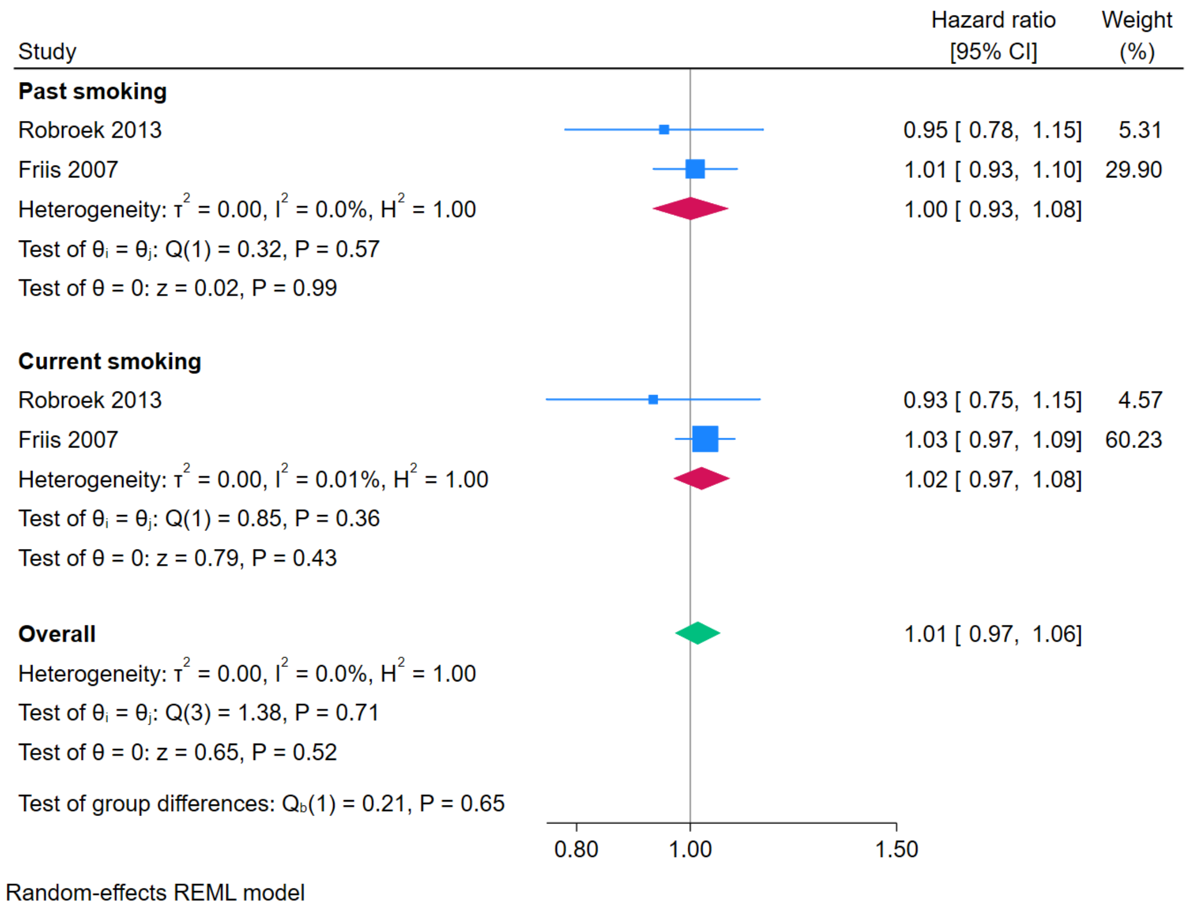

Figure S7: The effect of smoking on voluntary early old-age retirement.

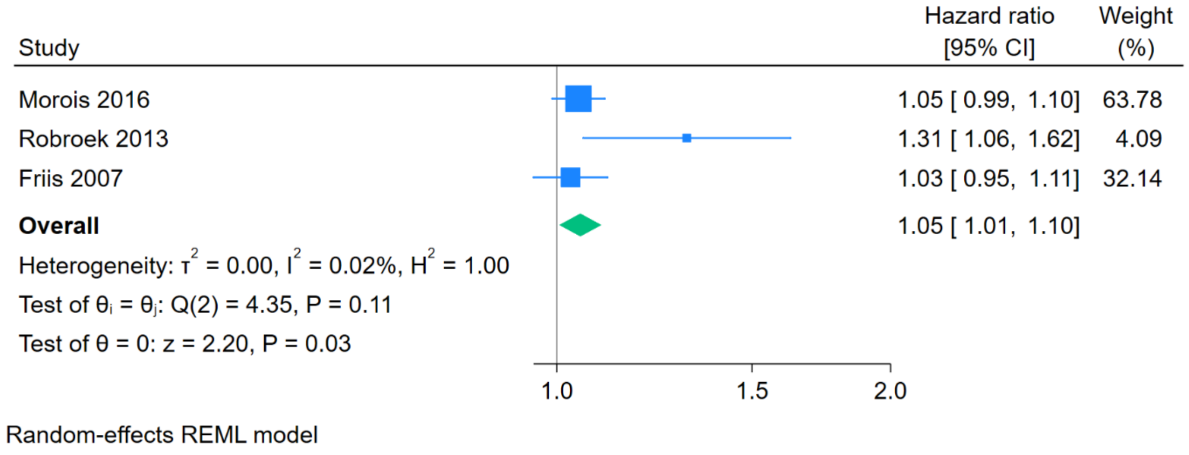

Figure S8: The effect of alcohol consumption on voluntary early old-age retirement.

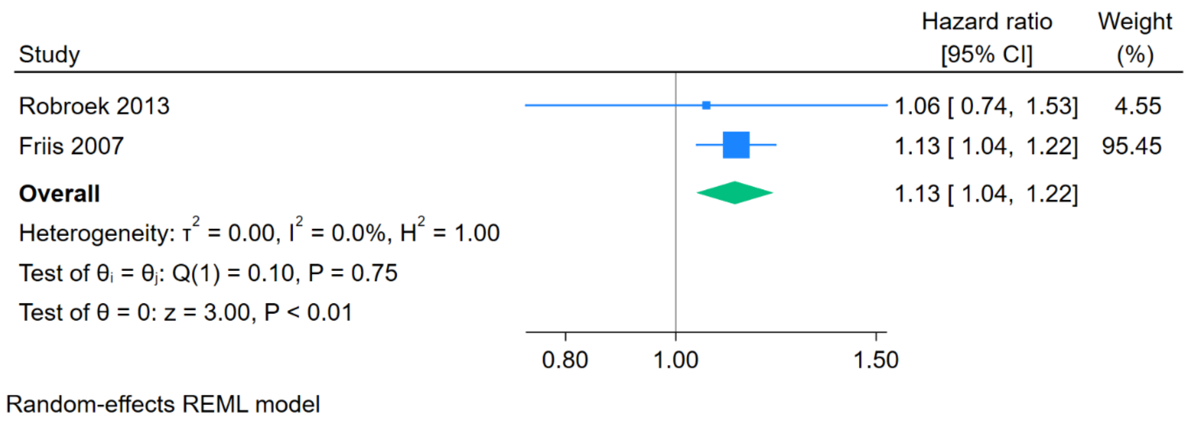

Figure S9: The effect of moderate or vigorous physical activity on voluntary early old-age retirement.

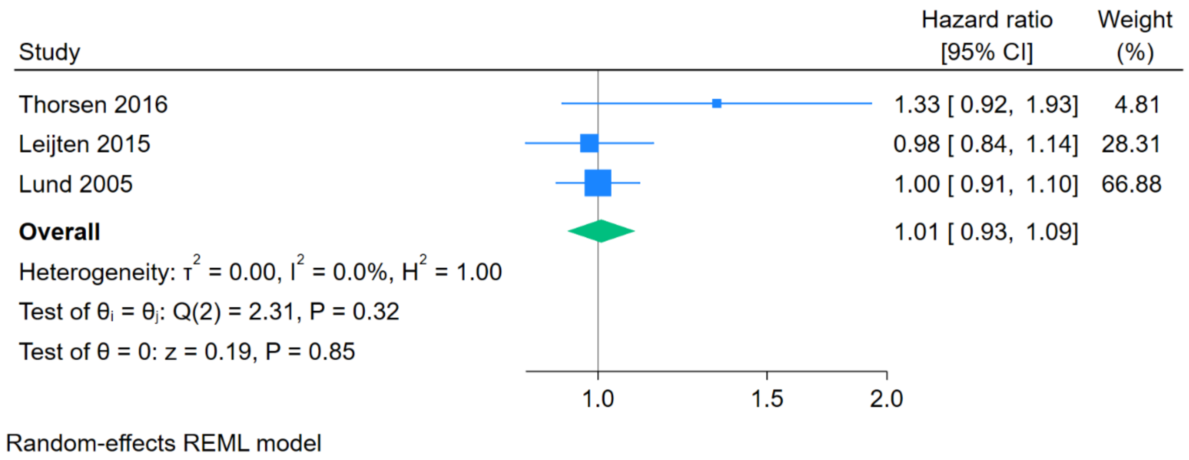

Figure S10: The effect of high emotional demands on voluntary early old-age retirement.

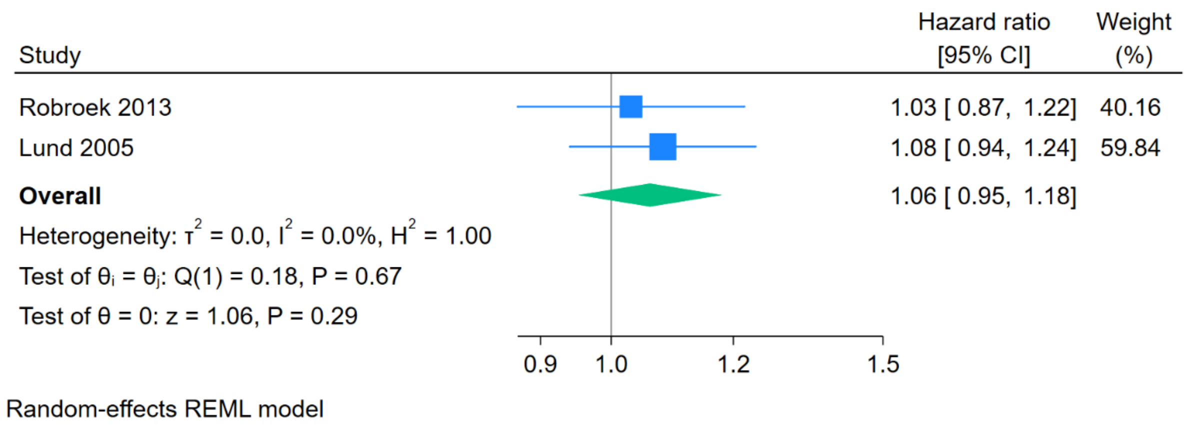

Figure S11: The effect of low job rewards on voluntary early old-age retirement.

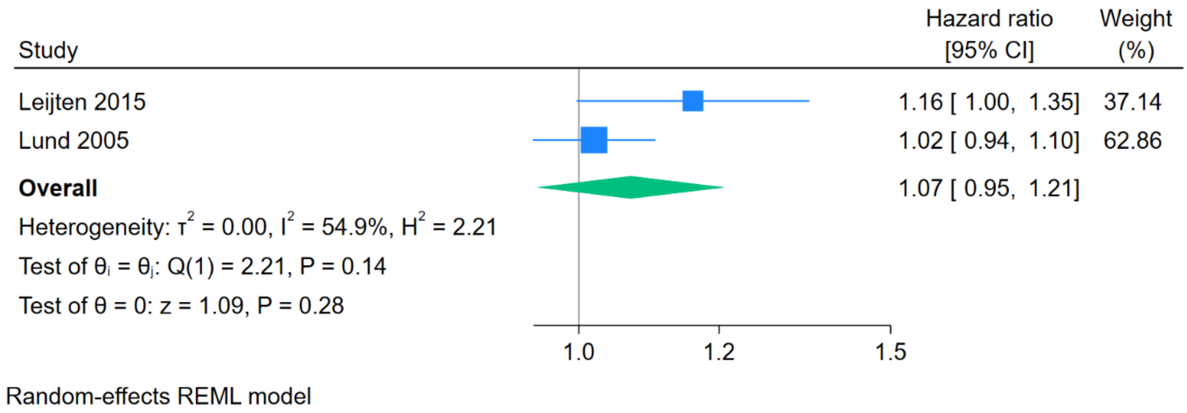

Figure S12: The effect of low social support on voluntary early old-age retirement.

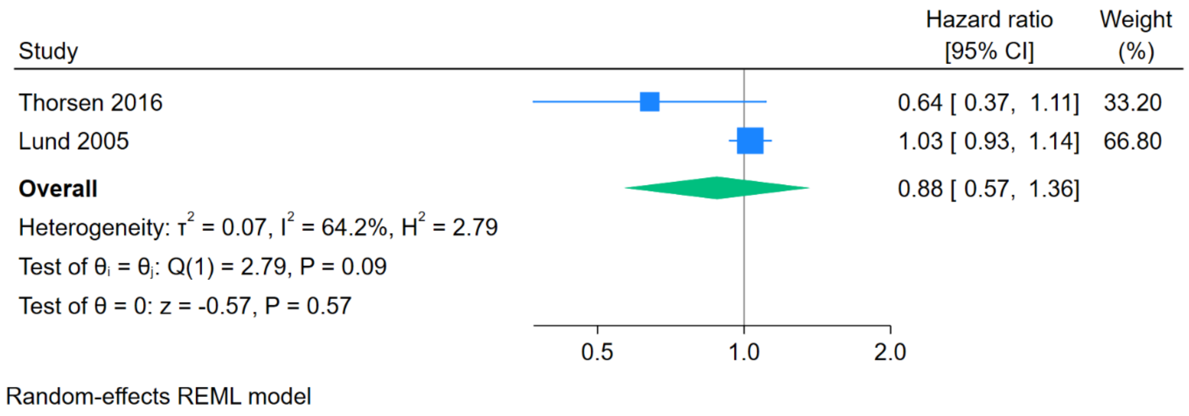

Figure S13: The effect of low predictability at work on voluntary early old-age retirement.

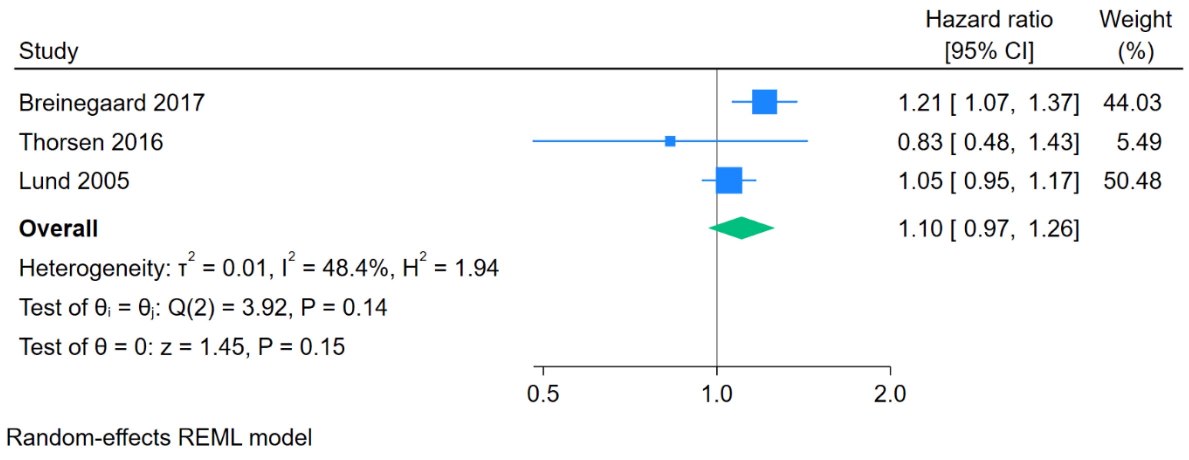

Figure S14: The effect of low quality of management on voluntary early old-age retirement.

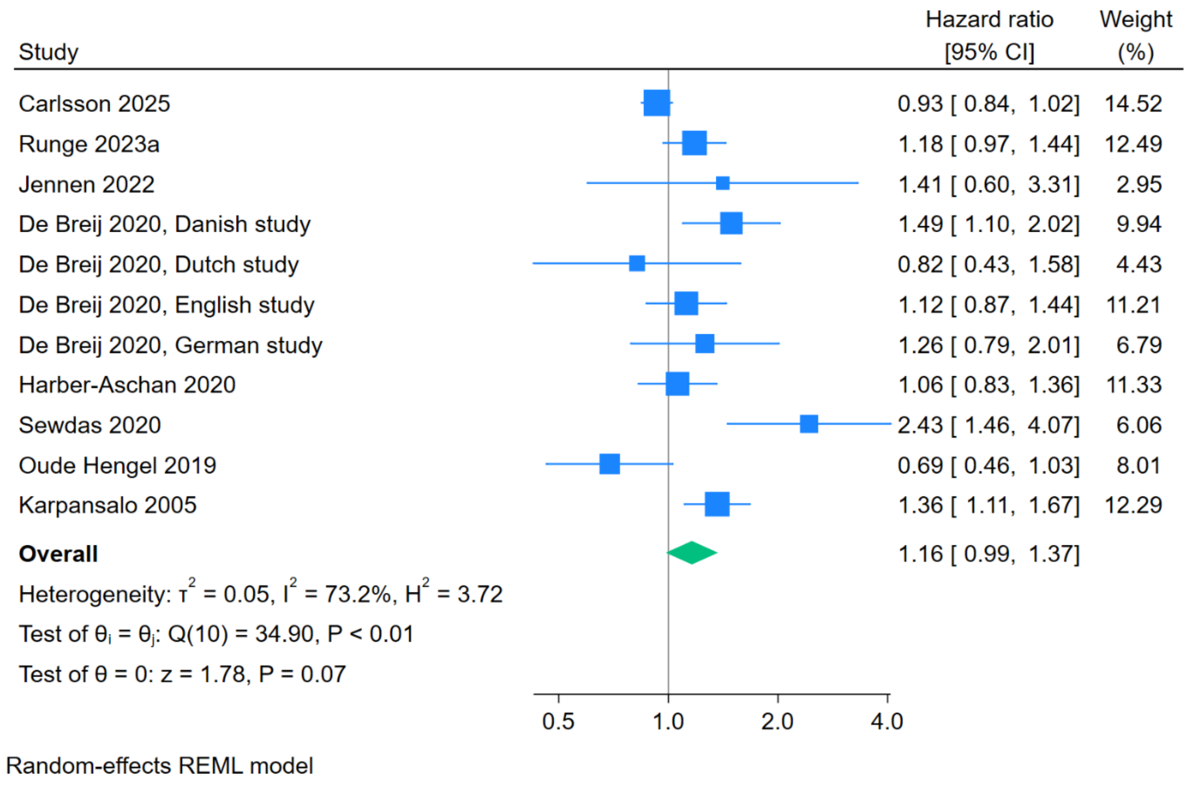

Figure S15: The effect of mental health conditions on voluntary early old-age retirement.

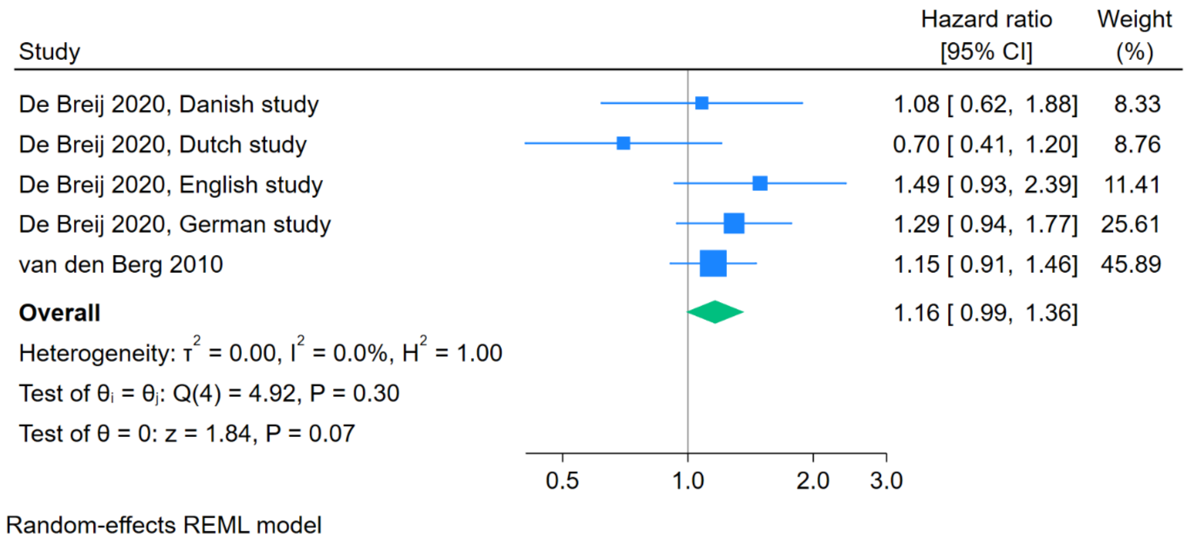

Figure S16: The effect of activity limitations on voluntary early old-age retirement.

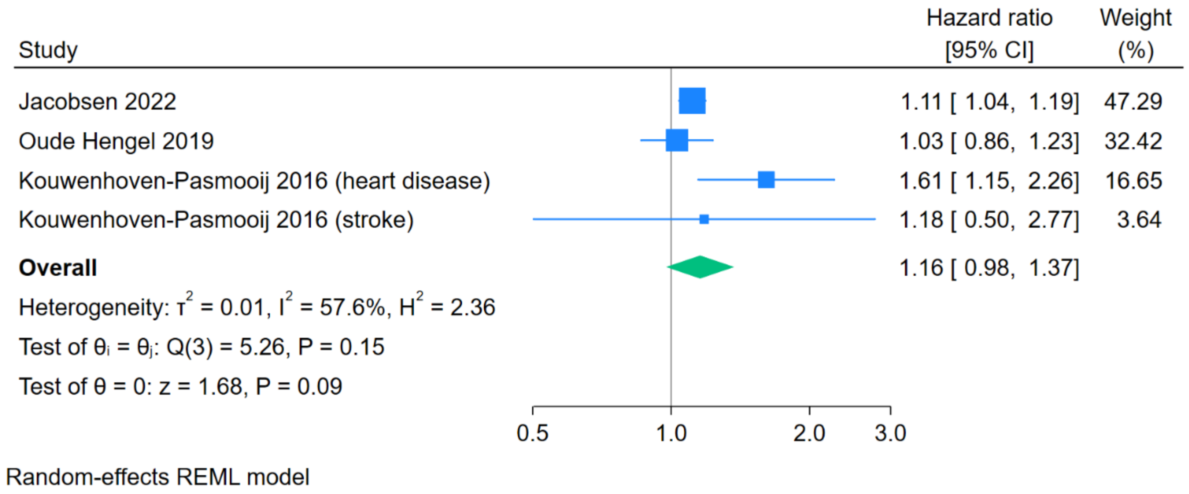

Figure S17: The effect of cardiovascular disease on voluntary early old-age retirement.

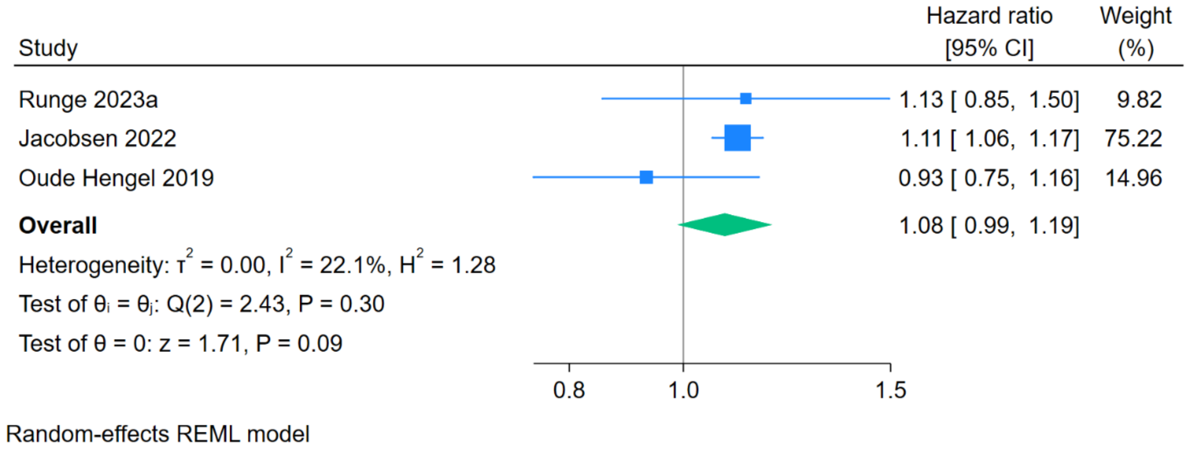

Figure S18: The effect of respiratory disease on voluntary early old-age retirement.

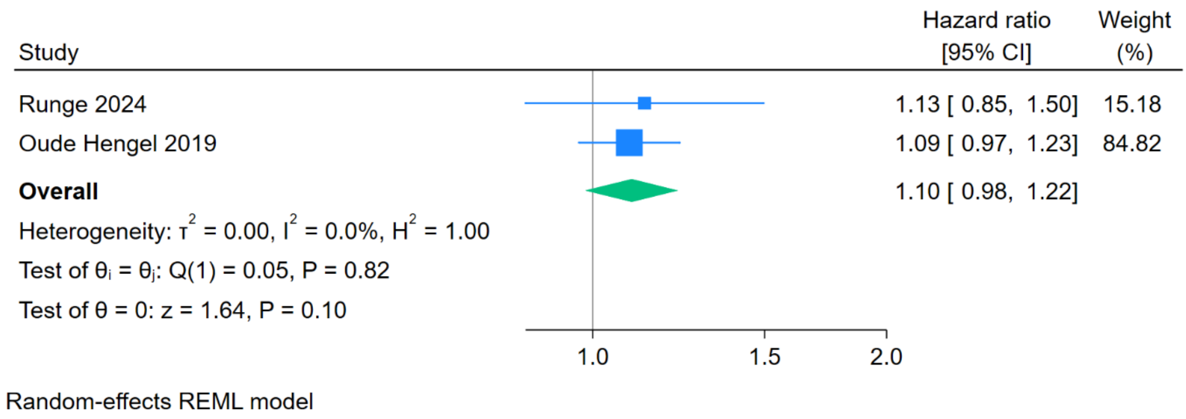

Figure S19: The effect of musculoskeletal disorders on voluntary early old-age retirement.

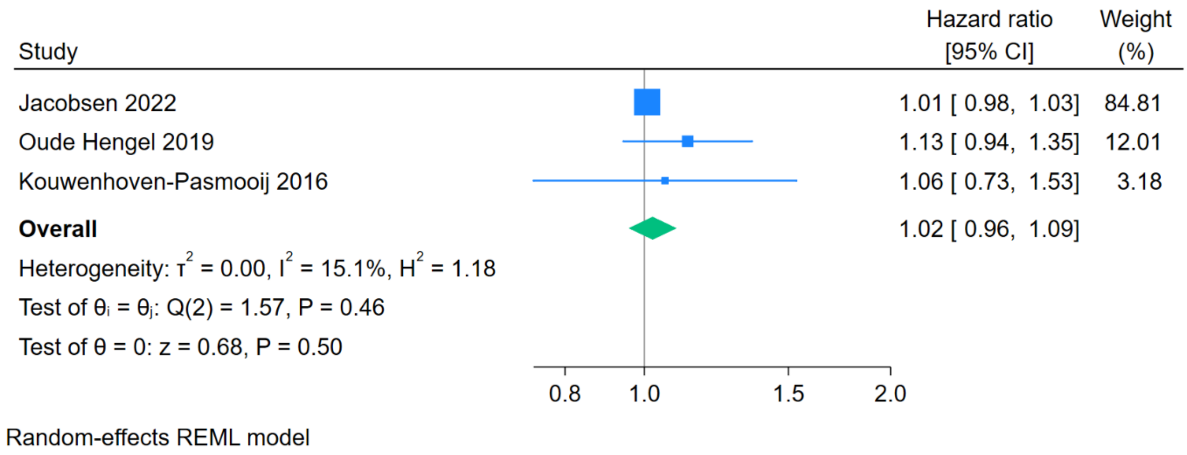

Figure S20: The effect of diabetes on voluntary early old-age retirement.

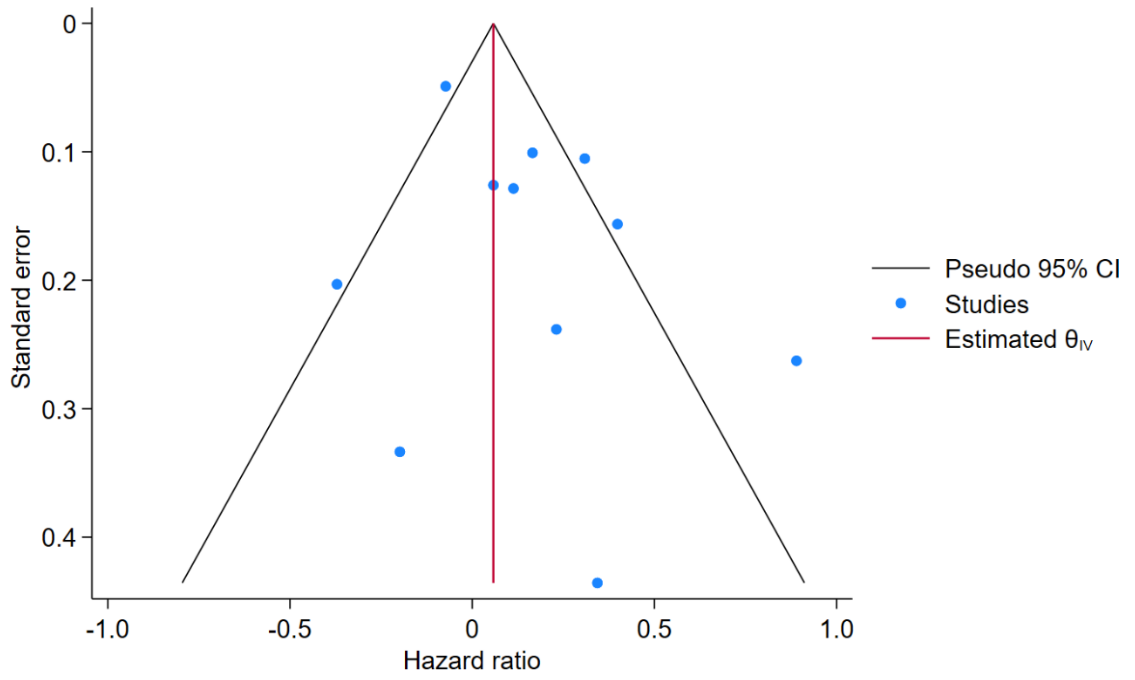

Figure S21: Funnel plot for studies examining the association between mental health conditions and voluntary early old-age retirement.

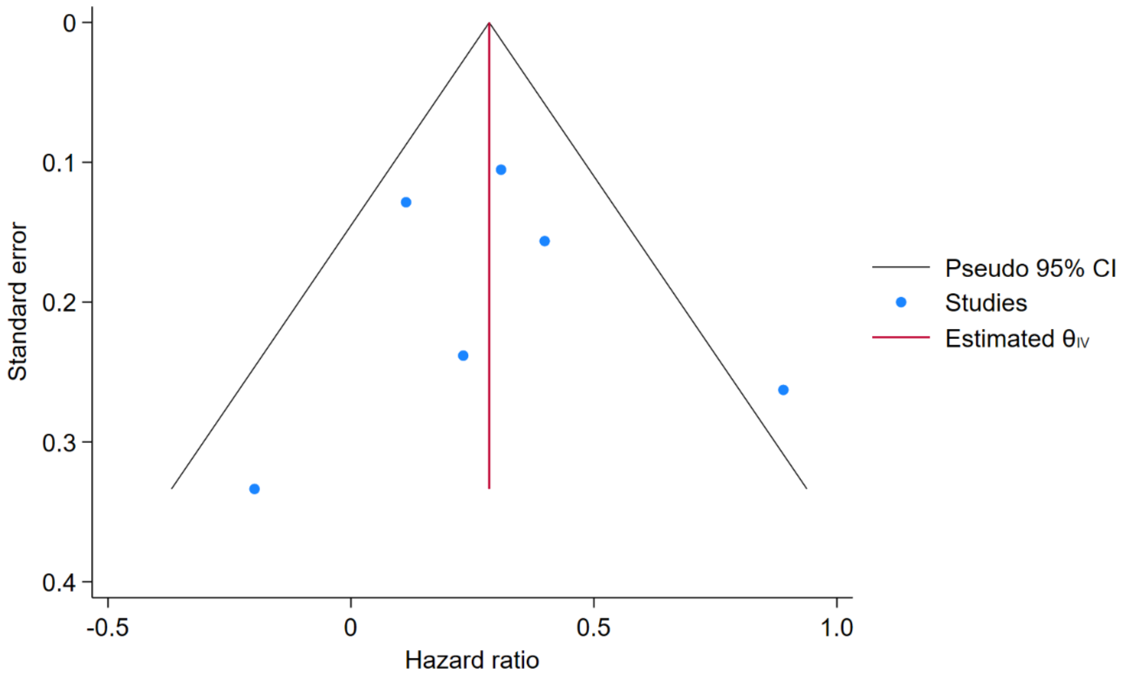

Figure S22: Funnel plot for studies examining the association between depressive symptoms and voluntary early old-age retirement.

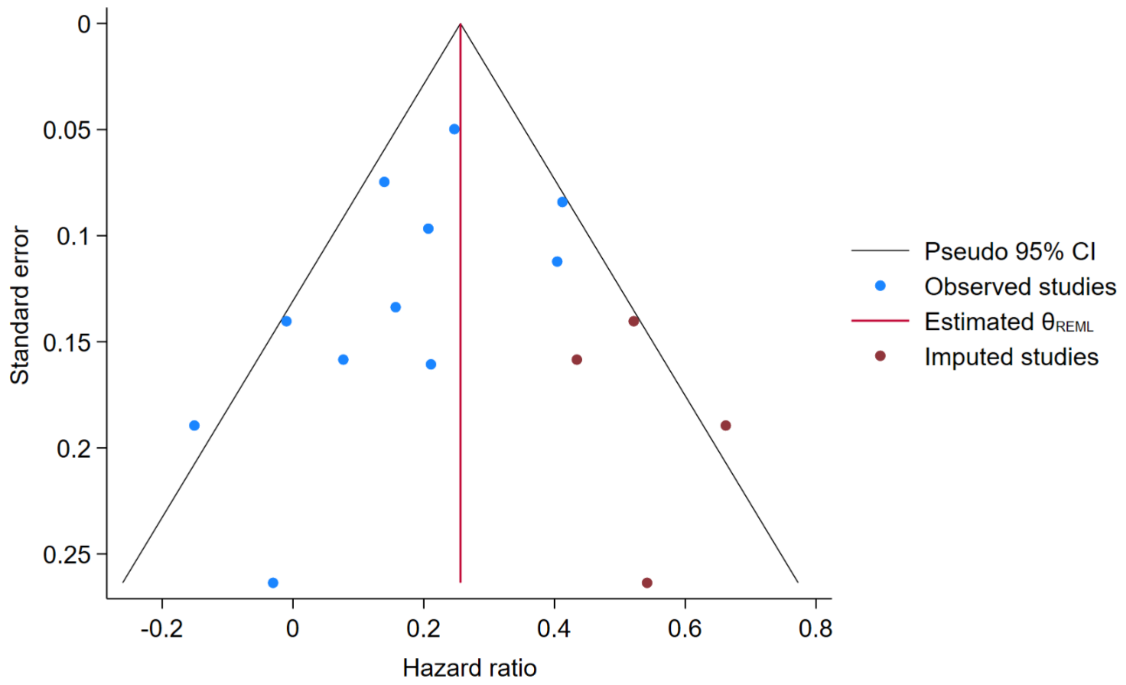

Figure S23: Funnel plot of the observed and imputed studies on the association between self-rated general health and voluntary early old-age retirement.

Supplementary Table S1: Quality assessment checklist

Type of bias	Criteria definition	Classification (potential for bias)
Selection bias	Sampling method of the study population, representativeness (response rate, difference between responders and non-responders, investigate and control of variables in case of difference between responders and non-responders)	**Low:** Target population defined as representative of the general population or subgroup of the general population (specific age group, women, men, specific geographic area, and specific occupational group) and response rate is 80% or more.<br>**Moderate:** Target population defined as somewhat representative of the general population, a restricted subgroup of the general population, response rate 60%-79%.<br>**High:** Target population defined as "self-referred" or "self-selected"/ volunteers, response rate less than 60%.
Performance bias	Valid and reliable assessment of exposure<br>Assessors blinded to outcome status	**Low:** Exposure was objectively assessed.<br>**Moderate:** A validated questionnaire was employed, or the assessment was reliant on self-reporting.<br>**High**: An appraisal of exposure does not adequately differentiate between exposed and unexposed individuals.
Detection bias	Standard method for outcome assessment<br>The assessor of outcome blinded to exposure status	**Low:** Register-based unemployment.<br>**Moderate:** Self-reported unemployment.
Confounding	Matching two groups<br>Stratification<br>Statistical analysis	**Low:** Controlled for most potential confounding factors, including age and sex.<br>**Moderate:** Controlled for several, but not the majority, of potential confounding factors.<br>**High:** Controlled for only a few confounding factors.
Attrition bias	Withdrawals and drop-out rates<br>Size of missing data	**Low:** Follow up participation rate of 80% or higher or missing data on less than 20%.<br>**Moderate:** Follow up participation rate of 60% –79%, or missing data on 20%–40%.<br>**High:** Follow up participation rate of less than 60%, or missing data on more than 40%.

Supplementary Table S2: The studies included in the review are listed in chronological order based on the most recent report of each study.

No. of studies	First author and year of publication	Country	Follow-up time (years)	Study population	Age range at baseline	Sex	Sample size (in analysis)	Exposure	Retirement	Results	Adjustment for other covariates
1	Carlsson 2025 (29)	Sweden	Up to 12 years	Individuals born from 1951 to 1953, who completed their compulsory military service in the years 1969–1972 and were alive in 2006.	52–54 years	Men	130 570	Register-based inpatient common mental disorders (depressive disorders, anxiety disorders, and stress-related disorders) and alcohol-related morbidity (alcohol dependency, alcoholic liver disease, and toxic effect of alcohol)	Register based early old-age retirement	Early old-age retirement with income. RR 0.93 (95% CI 0.85–1.03) for common mental disorders and 0.87 (95% CI 0.79–0.96) for alcohol-related morbidity.<br><br>Early old-age retirement without income. RR 1.01 (95% CI 0.94–1.08) for common mental disorders and 1.06 (95% CI 0.99–1.13) for alcohol-related morbidity.	Unadjusted
1	Carlsson 2024 (28)	Sweden	4 years	Individuals born from 1951 to 1953, who completed their compulsory military service in the years 1969–1972 and were alive in 2006.	53–55 years	Men	115 998	Physical workload and job control during 2005 based on job exposure matrices.<br><br>Physical workload and job control were dichotomised using the highest 25% of physical workload and the lowest 25% of job control.	Register based early old-age retirement	Early old-age retirement without income. RR 1.11 (1.08–1.13) for high physical workload and 1.04 (1.02–1.06) for low job control.<br><br>Early old-age retirement with income. RR 0.93 (0.90–0.96) for high physical workload and 1.14 (1.11–1.17) for low job control.	Unadjusted
1	Carlsson 2023 (23)	Sweden	4 years	Individuals born from 1951 to 1953, who	52–54 years	Men	130 768	Educational level.	Register based early old-age retirement	Early old-age retirement without income.	Childhood factors (parental education, parental occupation,

No. of studies	First author and year of publication	Country	Follow-up time (years)	Study population	Age range at baseline	Sex	Sample size (in analysis)	Exposure	Retirement	Results	Adjustment for other covariates
				completed their compulsory military service in the years 1969–1972 and were alive in 2006.				Educational duration was categorized as follows: up to 9 years for primary education, 10 to 11 years for two years of upper secondary school, 12 years for three years of upper secondary school, 13 to 14 years for two years of university education, and 15 years or more for three or additional years of university education.		HR 1.32 (95% CI 1.27–1.37) for individuals with 13-14 years of education, 1.45 (95% CI 1.39–1.51) for those with 12 years, 1.46 (95% CI 1.40–1.51) for 10-11 years, and 1.53 (95% CI 1.47–1.59) for those with nine years or less, compared to individuals with 15 or more years of education.<br><br>Early old-age retirement with income.<br><br>HR 1.18 (95% CI 1.12–1.24) for individuals with 13-14 years of education, 1.12 (95% CI 1.06–1.18) for those with 12 years, 1.24 (95% CI 1.18–1.29) for 10-11 years, and 1.27 (95% CI 1.21–1.34) for those with nine years or less, compared to individuals with 15 or more years of education.<br><br>Estimated HR 1.214 (95% CI 1.180–1.249) for 12 years or less education compared to individuals with 15 or more years of education.	and crowed housing), and late adolescence factors (cognitive ability, stress resilience, BMI ≥ 25 kg/m^2, muscle strength, cardiorespiratory fitness, psychiatric diagnoses, and musculoskeletal diagnoses)

No. of studies	First author and year of publication	Country	Follow-up time (years)	Study population	Age range at baseline	Sex	Sample size (in analysis)	Exposure	Retirement	Results	Adjustment for other covariates
										Estimated HR 1.11376 (95% CI 1.08256–1.145862) for 12 years or less education compared to individuals with 13 or more years of education.	
2	Almroth 2024 (27)	Sweden	3 years	The general working population (two subsamples of Swedish Work, Illness, and labor-market Participation [SWIP] cohort).<br><br>Cohort 1 included individuals aged 60 or 61 years in 2005 and Cohort 2 included individuals aged 60 or 61 years in 2012.	60–61 years	Both.<br><br>46% of Cohort 1 and 47% of Cohort 2 were women.	362 361 (N = 186 145 for Cohort 1 and N = 176 216 for Cohort 2)	Register based years of education.<br><br>Level of education was classified into three groups: primary (≤9 years), secondary (10–12 years) and tertiary (≥13 years).	Register based early old-age retirement	Early old-age retirement with income<br><br>Cohort 1<br>Men.<br>HR 0.82 (95% CI 0.79–0.86) for secondary education and 0.70 (95% CI 0.67–0.74) for primary education compared to tertiary education.<br><br>Women.<br>HR 0.69 (95% CI 0.66–0.72) for secondary education and 0.51 (95% CI 0.48–0.54) for primary education compared to tertiary education.<br><br>Estimated HR 0.696 (95% CI 0.679–0.712) for 12 years or less education compared to 13 years or higher education for both sexes combined.<br><br>Cohort 2<br>Men.<br>HR 1.16 (95% CI 1.11–1.21) for secondary education	Age, unemployment days, and sickness absence days

No. of studies	First author and year of publication	Country	Follow-up time (years)	Study population	Age range at baseline	Sex	Sample size (in analysis)	Exposure	Retirement	Results	Adjustment for other covariates
										and 1.26 (95% CI 1.20–1.32) for primary education compared to tertiary education.<br><br>Women.<br>HR 1.05 (95% CI 1.00–1.09) for secondary education and 0.85 (95% CI 0.79–0.91) for primary education compared to tertiary education.<br><br>Estimated HR 1.108 (95% CI 1.081–1.135) for 12 years or less education compared to 13 years or higher education for both sexes combined.	
										Early old-age retirement without income<br><br>Cohort 1<br>Men.<br>HR 1.15 (95% CI 1.10–1.20) for secondary education and 1.09 (95% CI 1.04–1.14) for primary education compared to tertiary education.<br><br>Women.<br>HR 1.15 (95% CI 1.09–1.20) for secondary education and 1.22 (95% CI 1.16–1.29) for primary education compared to tertiary education.	

No. of studies	First author and year of publication	Country	Follow-up time (years)	Study population	Age range at baseline	Sex	Sample size (in analysis)	Exposure	Retirement	Results	Adjustment for other covariates
										Cohort 2<br>Men.<br>HR 1.24 (95% CI 1.19–1.29) for secondary education and 1.26 (95% CI 1.20–1.31) for primary education compared to tertiary education.<br><br>Women.<br>HR 1.37 (95% CI 1.31–1.42) for secondary education and 1.54 (95% CI 1.47–1.62) for primary education compared to tertiary education.	
3	Runge 2024 (8) & Runge 2023 (60)	The Netherlands	Median 4.3 (SD 1.9) years	A representative sample of the general population	40–64 years.<br><br>Mean age 48.1 ± 5.7 years	Both. 54.2% were women.	55 016	Educational level, partner status, perceived general health, metabolic syndrome, diabetes, and chronic conditions.<br><br>Perceived general health was evaluated using a five-point scale.<br><br>Metabolic syndrome was defined as having at least three out of five components.<br><br>Self-reported chronic diseases.	Self-reported early old-age retirement at follow-up	HR 1.38 (95% CI 1.36–1.39) for 1-year increase in age, 0.52 (95% CI 0.40–0.67) for not married or partnered vs. married or partnered, and 1.06 (95% CI 0.90–1.24) for male sex.<br><br>Estimated HR 0.9434 (95% CI 0.8037–1.1073) for women compared to men.<br><br>HR 0.88 (95% CI 0.74–1.05) for medium education and 0.89 (95% CI 0.75–1.07) for low education compared with high education.<br><br>Estimated HR 0.885 (95% CI 0.781–1.002)	Age, sex, marital status, education, occupational class, working hours, perceived general health, metabolic syndrome, musculoskeletal disorders, pulmonary disease, cancer, and psychiatric disorders

No. of studies	First author and year of publication	Country	Follow-up time (years)	Study population	Age range at baseline	Sex	Sample size (in analysis)	Exposure	Retirement	Results	Adjustment for other covariates
										for 12 years or less education compared to higher level.<br><br>HR 0.73 (95% CI 0.62–0.87) for low-skilled white-collar, 0.61 (95% CI 0.47–0.79) for high-skilled blue-collar, and 0.47 (95% CI 0.35–0.64) for low-skilled blue-collar occupations compared with high-skilled white-collar occupations.<br><br>Estimated HR 0.546 (95% CI 0.448–0.665) for low occupational class, combining high-skilled blue-collar and low-skilled blue-collar occupations.<br><br>HR 1.13 (95% CI 0.85–1.49) for musculoskeletal disorders, 0.77 (95% CI 0.61–0.97) for pulmonary disease, 1.12 (95% CI 0.89–1.41) for cancer and 1.18 (95% CI 0.97–1.44) for psychiatric disorders.<br><br>HR 0.99 (95% CI 0.75–1.30) for less than good perceived general health and 1.10 (95% CI 0.94–1.29) for metabolic syndrome.	

No. of studies	First author and year of publication	Country	Follow-up time (years)	Study population	Age range at baseline	Sex	Sample size (in analysis)	Exposure	Retirement	Results	Adjustment for other covariates
										There was no dose–response relationship between the number of metabolic syndrome components and the risk of early old-age retirement. (60)	
4	Hansen 2022 (32)	Norway	Up to 16 years	Statistics Norway database	50–66 years.<br><br>Mean age 55.0 ± 3.8 years	Both.<br><br>48.4% were women.	7 585 730 observations.<br><br>The number of participants was not reported. It can be around a million.	Marital status, immigrant status, income, long-term sickness absence, and partner retirement.<br><br>Group 1 included immigrants from EU/EFTA, North America, Australia, and New Zealand.<br><br>Group 2 included immigrants from non-EU/EFTA European countries, Asia, Turkey, Africa, South and Central America, Oceania not including Australia and New Zealand.	Register based early old-age retirement	Men.<br><br>HR 0.94 (SE 0.03) for unmarried and 0.92 (SE 0.01) for divorced, separated or widow(er) compared to married or registered partner<br><br>Estimated HR 0.922 (95% CI 0.905–0.939) for unmarried, divorced, separated or widow(er) compared to married or registered partner.<br><br>HR 1.01 (SE 0.01) for primary school and for 0.88 (SE 0.01) for university/college compared to high school.<br><br>Estimated HR 1.1477 (1.1254–1.1705) for primary school and 1.1364 (1.1143–1.1589) for high school compared to university/college.<br><br>HR 0.67 (SE 0.03) for Group 1 immigrants and 0.62 (SE 0.67, authors' mistake, around 0.06 is correct)	Age and calendar year

No. of studies	First author and year of publication	Country	Follow-up time (years)	Study population	Age range at baseline	Sex	Sample size (in analysis)	Exposure	Retirement	Results	Adjustment for other covariates
										for Group 2 compared to natives.<br><br>HR 1.03 (SE 0.02) for log-term sickness absence at 47 years old.<br><br>HR 0.86 (SE 0.01) for log income.<br><br>HR 1.26 (SE 0.03) for partner retirement.<br><br>HR 1.09 (SE 0.02) for partner disability.<br><br>Women.<br><br>HR 0.85 (SE 0.03) for unmarried and 0.68 (SE 0.01) for divorced, separated or widow(er) compared to married or registered partner<br><br>Estimated HR 0.695 (95% CI 0.683–0.708) for unmarried, divorced, separated or widow(er) compared to married or registered partner.<br><br>HR 1.01 (SE 0.02) for primary school and for 1.07 (SE 0.02) for university/college compared to high school.<br><br>Estimated HR 0.9439 (0.9076–0.9817) for primary school and 0.9346 (0.8986–	

No. of studies	First author and year of publication	Country	Follow-up time (years)	Study population	Age range at baseline	Sex	Sample size (in analysis)	Exposure	Retirement	Results	Adjustment for other covariates
										0.9720) for high school compared to university/college.<br><br>HR 0.72 (SE 0.03) for Group 1 immigrants and 0.63 (SE 0.06) for Group 2 compared to natives.<br><br>HR 1.00 (SE 0.01) for long-term sickness absence at 47 years old.<br><br>HR 1.27 (SE 0.02) for log income.<br><br>HR 1.13 (SE 0.02) for partner retirement.<br><br>HR 0.98 (SE 0.02) for partner disability.<br><br>Both sexes combined.<br><br>Estimated HR 0.801 (95% CI 0.790–0.811) for unmarried, divorced, separated or widow(er) compared to married or registered partner.<br><br>Estimated HR 1.098 (1.085–1.112) for 12 years or less education compared to higher level.<br><br>Estimated HR 0.680 (95% CI 0.655–0.706) for immigrants compared to natives.	

No. of studies	First author and year of publication	Country	Follow-up time (years)	Study population	Age range at baseline	Sex	Sample size (in analysis)	Exposure	Retirement	Results	Adjustment for other covariates
5	Jacobsen 2022 (11)	Denmark	1	All employed Danish individuals with an unemployment insurance	60 years	Both.<br><br>47.6% were women.	627 278	Sex, education, income, diabetes, chronic obstructive pulmonary disease, and heart failure	Register based voluntary early retirement	OR 0.49 (95% CI 0.48–0.50) for men compared to women.<br><br>Estimated OR 2.041 (95% CI 1.999–2.083) for women compared to men.<br><br>Estimated RR 1.654 (95% CI 1.632–1.675) for women compared to men.<br><br>OR 0.75 (95% CI 0.74–0.76) for upper secondary education, 0.61 (95% CI 0.59–0.62) for tertiary education to bachelor or equivalent level, and 0.20 (95% CI 0.19–0.21) for masters/doctoral degree or equivalent compared to education up to lower secondary level.<br><br>Estimated OR 0.490 (95% CI 0.479–0.501) for tertiary or higher education compared to education up to lower secondary level.<br><br>Estimated OR 2.041 (95% CI 1.995–2.087) for education up to lower secondary level and 1.531 (95% CI 1.510–1.551) for upper secondary education compared to tertiary or higher education.	Sex, education, income, diabetes, chronic obstructive pulmonary disease, heart failure, and the number of comorbidities

No. of studies	First author and year of publication	Country	Follow-up time (years)	Study population	Age range at baseline	Sex	Sample size (in analysis)	Exposure	Retirement	Results	Adjustment for other covariates
										Estimated OR 1.650 (95% CI 1.631–1.669) for education up to upper secondary level compared to tertiary or higher education.<br><br>Estimated RR 1.439 (95% CI 1.428–1.451) for education up to upper secondary level compared to tertiary or higher education.<br><br>OR 1.33 (95% CI 1.27–1.39) for low income and 0.83 (95% CI 0.78–0.88) for high income compared to medium income.<br><br>OR 1.15 (95% CI 1.07–1.22) for chronic obstructive pulmonary disease.<br><br>Estimated RR 1.112 95% CI 1.053–1.162) for chronic obstructive pulmonary disease.<br><br>OR 1.15 (95% CI 1.05–1.25) for heart failure.<br><br>Estimated RR 1.112 95% CI 1.038–1.183) for heart failure.<br><br>OR 1.01 (95% CI 0.98–1.04) for diabetes.	

No. of studies	First author and year of publication	Country	Follow-up time (years)	Study population	Age range at baseline	Sex	Sample size (in analysis)	Exposure	Retirement	Results	Adjustment for other covariates
										Estimated RR 1.008 (95% CI 0.984–1.031) for diabetes.<br><br>OR 0.87 (95% CI 0.85–0.88) for healthy individuals compared to those with a chronic physical condition.<br><br>Estimated OR 1.1494 (95% CI 1.1296–1.1695) for a chronic physical condition.<br><br>Estimated RR 1.112 (95% CI 1.098–1.127) for a chronic physical condition.<br><br>Estimated RR 1.112 (95% CI 1.0388–1.1903) for a chronic physical condition based on the average standard errors of chronic obstructive pulmonary disease, heart failure, and diabetes.	
6	Jennen 2022 (61)	The Netherlands	6	Workers from 45 companies in different sectors	45–59.2 years	Both.<br><br>27.6% were women.	1755	Perceived general health, and chronic conditions.<br><br>Perceived general health was evaluated using a five-point scale.<br><br>Self-reported current chronic	Self-reported early old-age at follow-up	HR 0.79 (95% CI 0.53–1.18) for chronic physical condition, 1.41 (95% CI 0.60–3.31) for chronic mental condition, and 1.12 (95% CI 0.57–2.21) for comorbid chronic physical and mental conditions.<br><br>HR 0.97 (95% CI 0.58–1.63) for less	Age, sex, education, living alone, physically demanding work, psychological job demands, decision latitude, emotional demands, colleagues social support, supervisor social support, working hours, and perceived general health

No. of studies	First author and year of publication	Country	Follow-up time (years)	Study population	Age range at baseline	Sex	Sample size (in analysis)	Exposure	Retirement	Results	Adjustment for other covariates
								physical and mental conditions.		than good perceived general health.	
7-10	De Breij 2020 (62)	Denmark, Germany, United Kingdom, and the Netherlands.	Up to 15	A sample of general population.<br><br>Four studies: 1) Longitudinal Aging Study Amsterdam, 2) Danish Longitudinal Study of Ageing), 3) English Longitudinal Study of ageing, and 4) German Ageing Survey	50 years or older	Both	Danmark (N=4721), Germany (N=1203), United Kingdom (N=4508), and the Netherlands (N=1295)	Perceived general health, functional limitations, and depression.<br><br>Perceived general health was evaluated using a five-point scale.<br><br>To evaluate functional limitations, the studies employed various metrics. The Dutch and Danish studies utilized a set of six items, the English study applied five items, while the German study measured using 10 items from the SF-36 scale (such as walking and dressing).<br><br>For assessing depressive symptoms, the Dutch study incorporated 20 items, the English study 8 items, and the German study 15 items from the Centre for Epidemiologic Studies Depression Scale.	Self-reported early old-age retirement at follow-up	HRs for less than good perceived general health were 1.51 (95% CI 1.28–1.78) for Danmark, 1.08 (95% CI 0.79–1.47) for Germany, 1.15 (95% CI 1.00–1.34) for United Kingdom and 0.86 (95% CI 0.59–1.24) for the Netherlands.<br><br>HRs for functional limitations were 1.08 (95% CI 0.62–1.88) for Danmark, 1.29 (95% CI 0.94–1.77) for Germany, 1.49 (95% CI 0.93–2.40) for United Kingdom and 0.70 (95% CI 0.41–1.21) for the Netherlands.<br><br>HRs for depression were 1.49 (95% CI 1.10–2.03) for Danmark, 1.26 (95% CI 0.79–2.01) for Germany, 1.12 (95% CI 0.87–1.44) for United Kingdom and 0.82 (95% CI 0.43–1.59) for the Netherlands.	Age, sex, education, partner status, number of working hours, year, and region

No. of studies	First author and year of publication	Country	Follow-up time (years)	Study population	Age range at baseline	Sex	Sample size (in analysis)	Exposure	Retirement	Results	Adjustment for other covariates
								In the Danish study, data on depression relied on self-reports of physician-diagnosed depression or instances of depression occurring in the preceding year.			
11	Harber-Aschan 2020 (33)	Sweden	≥ 2 years	A random sample of general population	50–62 years	Both.<br><br>53% were women.	10 416	Sex, educational level, marital status, occupational class, financial strain, limiting long-standing illnesses and common mental disorders.<br><br>Common mental disorders were assessed using the 12-item General Health Questionnaire (score ≥4).<br><br>Data on limiting long-standing illness was based on self-reported health problems limiting the ability to work or do other daily activities.<br><br>Financial strain was defined as borrowing money from	Register based early old-age retirement at follow-up	HR 0.71 (95% CI 0.62–0.80) for single, divorced or widowed compared with married or in register-based partnership.<br><br>HR 0.79 (95% CI 0.65–0.96) for born outside Sweden and 1.05 (95% CI 0.92–1.19) for female sex.<br><br>HR 1.42 (95% CI 1.15–1.75) for primary education and 1.43 (95% CI 1.23–1.66) for secondary education compared with higher education.<br><br>Estimated HR 1.427 (95% CI 1.263–1.612) for 12 years or less education compared to higher level.<br><br>HR 1.03 (95% CI 0.88–1.20) for intermediate non-manual occupations, 0.78 (95% CI 0.64–0.95) for low non-	Age, sex, education, marital status, country of birth, occupational class, financial strain, employment condition and presence of physical and mental condition

No. of studies	First author and year of publication	Country	Follow-up time (years)	Study population	Age range at baseline	Sex	Sample size (in analysis)	Exposure	Retirement	Results	Adjustment for other covariates
								family/friends to afford food or rent in the past year.		manual and 0.60 (95% CI 0.48–0.76) for manual compared with high non-manual occupations.<br><br>Estimated HR 0.926 (95% CI 0.820–1.047) for intermediate occupational class, combining intermediate non-manual and low non-manual occupations.<br><br>HR 0.90 (95% CI 0.64–1.27) for financial strain.<br><br>HR 0.90 (95% CI 0.76–1.07) for long-standing illness only, 1.06 (95% CI 0.83–1.36) for a common mental disorder only, and 1.10 (95% CI 0.79–1.53) for comorbid limiting long-standing illnesses and common mental disorder compared with those without both conditions.	
12	Sewdas 2020 (63)	Denmark	4 years	The Danish National Working Environment Survey (DANES)	56–64 years	Both.<br><br>50.6% were women.	1861	Physically demanding work, work-family conflict, self-rated general health, chronic disease, and depressive symptoms.<br><br>Physically demanding work	Register based early old-age retirement	HR 2.09 (95% CI 1.39–3.13) for high physically demanding work among workers without chronic diseases and 1.84 (95% CI 1.37–2.48) among workers with a chronic disease.	Age, sex, education and mode of data collection

No. of studies	First author and year of publication	Country	Follow-up time (years)	Study population	Age range at baseline	Sex	Sample size (in analysis)	Exposure	Retirement	Results	Adjustment for other covariates
								was assessed using a single question.<br><br>Three items were used to assess work–family conflict, and analyzed as a continuous variable<br><br>Information on chronic disease was based on self-reported ever physician-diagnosed disease.<br><br>Self-rated general health was based on a single question and recoded as 0, 0.25, 0.5, 0.75 and 1.0 for excellent, very good, good, fair, and poor, respectively, and analyzed as a continuous variable.<br><br>Depressive symptoms were assessed by the 12-item Major Depression Inventory Scale.		Estimated HR 1.923 (95% CI 1.514–2.444) for high physically demanding work among all workers (with or without a chronic disease).<br><br>HR 1.32 (95% CI 0.76–2.29) for work-family conflict among workers without chronic diseases and 1.59 (95% CI 1.11–2.27) among workers with a chronic disease.<br><br>HR 1.18 (95% CI 1.02–1.37) for a chronic disease.<br><br>HR 2.56 (95% CI 1.27–5.16) for poor self-rated heath among workers without chronic diseases and 2.15 (95% CI 1.37–3.37) among workers with a chronic disease.<br><br>HR 4.22 (95% CI 1.47–12.11) for depressive symptoms among workers without chronic diseases and 2.05 (95% CI 1.14–3.71) among workers with a chronic disease.<br><br>Estimated HR 2.435 (95% CI 1.455–4.075) for depressive symptoms among all workers (with or	

No. of studies	First author and year of publication	Country	Follow-up time (years)	Study population	Age range at baseline	Sex	Sample size (in analysis)	Exposure	Retirement	Results	Adjustment for other covariates
										without a chronic disease).	
12	Thorsen 2016 (64)	Denmark	4 years	The Danish National Working Environment Survey. It included 3 samples: 1) a representative sample of the general population, 2) a representative sample of employees aged 50 years and older, and 3) a representative sample of private companies with 10–500 employees	56–64 years.<br><br>Mean age 59 years	Both.<br><br>50% were women.	1876	Psychosocial factors.<br><br>Emotional demands, influence at work, possibilities of development, organizational justice, leadership quality, and predictability were each assessed by 2 items.<br><br>Job satisfaction, work pace, quantitative demands, role conflicts, role clarity, age discrimination, recognition from management, trust in management, social community at work, and trust between colleagues were each assessed by a single item.	Register based early old-age retirement	HR 3.33 (95% CI 2.36–4.70) for low job satisfaction.<br><br>HR 1.30 (95% CI 0.90–1.88) for low influence at work.<br><br>HR 1.98 (95% CI 1.20–3.27) for low possibilities of development.<br><br>HR 1.03 (95% CI 0.70–1.53) for work pace.<br><br>HR 1.10 (95% CI 0.78–1.55) for quantitative demands.<br><br>HR 1.33 (95% CI 0.92–1.93) for emotional demands.<br><br>HR 1.03 (95% CI 0.70–1.51) for role conflicts.<br><br>HR 1.19 (95% CI 0.76–1.88) for low role clarity.<br><br>HR 1.58 (95% CI 1.10–2.28) for age discrimination.<br><br>HR 1.69 (95% CI 1.13–2.52) for low recognition from management.	Sex, socioeconomic status, physical strain in the job, cohabitation, shift and night work, part-time job, sample, data collection method, mental health, self-rated health, and all psychosocial factors

No. of studies	First author and year of publication	Country	Follow-up time (years)	Study population	Age range at baseline	Sex	Sample size (in analysis)	Exposure	Retirement	Results	Adjustment for other covariates
										HR 1.18 (95% CI 0.64–2.20) for low organizational justice.<br><br>HR 1.15 (95% CI 0.70–1.91) for poor trust in management.<br><br>HR 0.83 (95% CI 0.48–1.43) for poor leadership quality.<br><br>HR 0.64 (95% CI 0.37–1.11) for poor predictability.<br><br>HR 0.98 (95% CI 0.62–1.56) for poor social community at work.<br><br>HR 0.73 (95% CI 0.50–1.08) for poor trust between colleagues.	
13	Oude Hengel 2019 (65)	The Netherlands	7	A sample of working and non-working individuals	45–64 years.<br><br>Mean age 53.8 ± 5.3 years	Both.<br><br>42.7% were women	9160	Self-reported current chronic diseases.	Register based early old-age retirement	HR 1.03 (95% CI 0.86–1.23) for cardiovascular disease, 1.13 (95% CI 0.94–1.35) for diabetes, 0.93 (95% CI 0.73–1.18) for digestive disease, 1.09 (95% CI 0.97–1.23) for musculoskeletal disorder, 0.69 (95% CI 0.46–1.02) for psychological disorder, and 0.93 (95% CI 0.74–1.15) for respiratory disease.<br><br>HR 1.05 (95% CI 0.95–1.18) for a single	Age, sex, educational level, marital status, and other chronic diseases

No. of studies	First author and year of publication	Country	Follow-up time (years)	Study population	Age range at baseline	Sex	Sample size (in analysis)	Exposure	Retirement	Results	Adjustment for other covariates
										chronic condition and 1.03 (95% CI 0.90–1.18) for multiple chronic conditions compared with those without chronic illnesses.	
14	Stynen 2019 (72)	The Netherlands	4 years	Employees from 45 Dutch companies and organizations (the Maastricht Cohort Study)	45–59 years.	Both.<br><br>27.3% were women.	2312	Need for recovery after work.<br><br>The need for recovery after work was assessed using an 11-item subscale from the Dutch Questionnaire on the Experience and Evaluation of Work (QEEW). Scores ranged from 0 to 11 and were dichotomized, with a score of 6 or higher indicating a significant need for recovery. Additionally, the continuous scores were converted to a scale of 0 to 100.	Self-reported early old-age retirement	HR 1.22 (95% CI 0.78–1.90) for elevated need for recovery after work (score 6 or higher vs. score <6).<br><br>HR 1.01 (95% CI 1.00–1.01) for 1-umit increase in continuous variable (score 0 to 100).	Age, sex, education, number of working hours per week, work schedule, psychological job demands, decision latitude, supervisor social support, self-perceived health, spousal retirement and attachment to work for financial resources
15	Sundstrup 2018 (31)	Denmark	4 to 6 years	A sample of middle-aged people living in the eastern parts of the country	49–63 years.<br><br>Mean age 54.3 ± 3.8 years	Both.<br><br>30.3% were women.	5076	Age and self-reported lifetime physical workload factors.<br><br>Physical work demands over a lifetime were categorized into	Register based early old-age retirement	HR 1.66 (95% CI 1.59–1.74) for 1-year increase in age.<br><br>HR 1.26 (95% CI 1.00–1.58) for moderate physical demands, 1.74 (95% CI 1.35–2.25) for high	Age, sex, socioeconomic position, psychosocial work environment (quantitative demands, emotional demands, decision authority, work

No. of studies	First author and year of publication	Country	Follow-up time (years)	Study population	Age range at baseline	Sex	Sample size (in analysis)	Exposure	Retirement	Results	Adjustment for other covariates
								four levels: low, moderate, high, and very high.<br><br>Specific workload factors included: 1) lifting or carrying heavy objects or individuals, 2) performing repetitive tasks for the majority of work hours, 3) using tools that produce hand vibrations,4) exerting force to pull or push heavy weights, 5) frequent twisting or bending of the back, 6) being in environments with dust from various sources such as cement, demolition sites, mineral fibers, wood, and biological materials, 7) welding smoke, 8) diesel fumes, and 9) noise.		demands and 1.57 (95% CI 1.13–2.17) for very high demands compared to low work demands (sedentary work).<br><br>Estimated HR 1.479 (95% CI 1.271–1.720) for moderate, high, or very high demands compared to low work demands.<br><br>Estimated HR 1.673 (95% CI 1.368–2.046) for high, or very high demands compared to low work demands.<br><br>HR 1.34 (95% CI 1.07–1.67) for more than 20 years exposure to noise, 1.40 (95% CI 1.11–1.78) for more than 20 years exposure to lifting/carrying of heavy loads and 1.52 (95% CI 1.12–2.05) for more than 20 years exposure to dust.<br><br>Other workload factors were not significantly associated with early old-age retirement.	pace, role conflicts, rewards, and possibilities for development), physical activity, body mass index, smoking, chronic diseases (back disease, cancer, and chronic depression or anxiety), previous long-term sickness absence
16	Breinegaard 2017 (9)	Denmark	1.7 years	Public service workers	58–64 years	Both.<br><br>75.2% were women.	3254	Psychosocial factors and organizational changes at work unit level.<br><br>Social	Register based early old-age retirement	HR 1.30 (95% CI 1.09–1.55) for lower work-unit mean social capital, 1.27 (95% CI 1.10–1.47) for work-unit lower mean organizational justice, and 1.21 (95% CI	Age, sex, marital status, occupation, medical condition, personal income, household income, and psychosocial or organizational factors

No. of studies	First author and year of publication	Country	Follow-up time (years)	Study population	Age range at baseline	Sex	Sample size (in analysis)	Exposure	Retirement	Results	Adjustment for other covariates
								capital was assessed using eight items, organizational justice was evaluated through six items, and quality of management was assessed using four items (all continuous variables).<br><br>Information regarding changes of management, merging, demerging, and relocation of units was gathered from heads of work units via an online questionnaire (all binary variables).		1.07–1.38) for work-unit lower mean quality of management.<br><br>HR 1.27 (95% CI 1.03–1.57) for change of management, 1.11 (95% CI 0.90–1.38) for merging units, 0.98 (95% CI 0.73–1.30) for demerging unit, and 1.10 (95% CI 0.87–1.40) for relocation of unit.	
17	de Wind 2017b (12)	Denmark	2 years	A representative sample of the working population who had unemployment insurance (the Danish Work Environment Cohort Study [DWECS])	49–64 year.<br><br>Median age 54.3 years	Both.<br><br>53.3% were women.	1167	Age, sex, socioeconomic status, physically demanding work, self-rated general health and quality of supervision.<br><br>Self-rated general health was assessed using a single item and classified into two groups: good health (very good and good responses) and poor health (fairly	Register based early old-age retirement	HR 1.04 (95% CI 1.02–1.06) for 1-year increase in age.<br><br>HR 1.49 (95% CI 1.26–1.76) for women vs. men.<br><br>HR 1.06 (95% CI 0.78–1.43) for lower professionals and managers, 1.23 (95% CI 0.96–1.68) for higher clerical services and sales workers, 1.45 (95% CI 1.08–1.96) for lower clerical services, sales,	Age, sex, socioeconomic status, physically demanding work, self-rated general health and quality of supervision

No. of studies	First author and year of publication	Country	Follow-up time (years)	Study population	Age range at baseline	Sex	Sample size (in analysis)	Exposure	Retirement	Results	Adjustment for other covariates
								good, poor, and very poor responses).<br><br>Physically demanding work was assessed using three items on sitting, squatting, pushing/pulling, and lifting.<br><br>Quality of supervision was assessed using the four items from the Copenhagen Psychosocial Questionnaire and split into two groups based on the median distribution.		technology and farm workers, 1.97 (95% CI 1.40–2.78) for skilled workers, and 1.86 (95% CI 1.39–2.47) for semi- and unskilled workers compared to higher professionals and managers.<br><br>Estimated HR 1.329 (95% CI 1.083–1.629) for intermediate occupational class, combining higher clerical services and sales workers and lower clerical services, sales, technology and farm workers compared to higher professionals and managers.<br><br>Estimated HR 1.905 (95% CI 1.528–2.374) for low occupational class, combining skilled workers and semi- and unskilled workers compared to higher professionals and managers.<br><br>Estimated HR 1.29 (95% CI 1.052–1.583) for intermediate occupational class and 1.8495 (95% CI 1.4838–2.305) for low occupational class compared to higher and lower professionals and managers.	

No. of studies	First author and year of publication	Country	Follow-up time (years)	Study population	Age range at baseline	Sex	Sample size (in analysis)	Exposure	Retirement	Results	Adjustment for other covariates
										HR 1.14 (95% CI 0.99–1.30) for 1-unit increase in physically demanding work.<br><br>HR 1.23 (95% CI 1.02–1.49) for poor vs. good self-rated health.<br><br>HR 1.04 (95% CI 0.90–1.21) for low vs. high quality of supervision.<br><br>There was no interaction between poor self-rated general health and low quality of supervision for the risk of early retirement.	
17	Lund 2005 (66)	Denmark	4	A sample of working population (the Danish Work Environment Cohort Study [DWECS])	57–62 years	Both	365	Age, sex, socioeconomic position, and physical and psychosocial factors.<br><br>Socioeconomic status was classified by a combination of job rank, title, and education into five groups: I) executives and academics, II) middle managers or having more than 3–4 years additional education, III) other white-collar	Register based early old-age retirement	OR 1.07 (95% CI 0.61–1.87) for cohabitation.<br><br>OR 1.14 (95% CI 0.54–2.44) for class II of socioeconomic status, 2.63 (95% CI 1.33–5.22) for class III, 3.49 (95% CI 1.48–8.24) for class IV and 2.39 (95% CI 1.12–5.09) for class V compared with class I of socioeconomic status.<br><br>OR 1.04 (95% CI 0.96–1.14) for low decision authority.	Age, sex, cohabitation, socioeconomic position, physical factors (controlled for psychosocial factors only), and psychosocial factors (controlled for physical factors)

No. of studies	First author and year of publication	Country	Follow-up time (years)	Study population	Age range at baseline	Sex	Sample size (in analysis)	Exposure	Retirement	Results	Adjustment for other covariates
								employees, IV) skilled blue-collar workers, and V) semi- or unskilled blue-collar workers.		OR 1.09 (95% CI 1.00–1.19) for low skill discretion.<br><br>OR 1.00 (95% CI 0.91–1.11) for high emotional demands.<br><br>OR 1.09 (95% CI 0.98–1.21) for high demands of bottling up emotions.<br><br>OR 1.08 (95% CI 0.97–1.21) for high job insecurity.<br><br>OR 1.02 (95% CI 0.94–1.10) for low social support.<br><br>OR 1.05 (95% CI 0.94–1.16) for poor management quality.<br><br>OR 1.08 (95% CI 0.94–1.24) for low reward in work.<br><br>OR 1.03 (95% CI 0.88–1.21) for low meaning in work.<br><br>OR 1.03 (95% CI 0.93–1.13) for low predictability in work.<br><br>OR 1.43 (95% CI 1.08–1.90) for high conflict in work.<br><br>OR 1.06 (95% CI 0.97–1.15) for intensive qualitative demands.	

No. of studies	First author and year of publication	Country	Follow-up time (years)	Study population	Age range at baseline	Sex	Sample size (in analysis)	Exposure	Retirement	Results	Adjustment for other covariates
										OR 1.20 (95% CI 1.03–1.40) for extreme bending or twisting of neck or back.<br><br>OR 1.08 (95% CI 0.93–1.26) for work with arms lifted or hands twisted.<br><br>OR 1.26 (95% CI 1.10–1.43) for working mainly standing or squatting.	
18	de Wind 2017a (67)	The Netherlands	2 years	A sample of working individuals (the Study on Transitions in Employment, Ability and Motivation [STREAM])	55–62 years	Both.<br><br>41.7% were women.	3171	Three-year trajectories of work engagement (the first, second and third years).<br><br>Work engagement was assessed by the 6-item Utrecht Work Engagement Scale, and current work ability was measured by a single item.	Self-reported early old-age retirement at the third or fourth year	OR 1.46 (95% CI 1.05–2.04) for steady low work engagement, 0.79 (95% CI 0.46–1.37) for decreasing work engagement, and 1.60 (95% CI 0.96–2.67) for increasing work engagement compared to steady high work engagement.	Age, sex, and education
18	de Wind 2015 (24)	The Netherlands	2 years	A sample of working individuals (STREAM)	58–62 years.<br><br>Mean age 59.7 ± 1.3 years	Both.<br><br>41% were women.	1862	Age, sex, education, household financial situation, physical workload, psychosocial factors, perceived physical and mental health, development of skills and knowledge, and	Self-reported early old-age retirement	Higher work ability (β -0.12) and a positive attitude of colleagues and supervisor about working until 65 years (β -0.23) were associated with lower risk of early retirement, while work engagement and age discrimination were not associated with early retirement.	Age, sex, and education

No. of studies	First author and year of publication	Country	Follow-up time (years)	Study population	Age range at baseline	Sex	Sample size (in analysis)	Exposure	Retirement	Results	Adjustment for other covariates
								life event (measured at baseline).<br><br>Work engagement, work ability, age discrimination, and attitude of coworkers and supervisors toward early retirement (measured at 1-year follow-up).<br><br>Work engagement was assessed by the 6-item Utrecht Work Engagement Scale, and current work ability was measured by a single item.<br><br>Age discrimination was assessed using three items about promotion, education and training, and development.		Low work ability and negative attitude of coworkers and supervisors toward working until age 65 mediated the associations of perceived poor health, job characteristics, development of skills and knowledge, and social factors with early old-age retirement.<br><br>High support from colleagues and supervisors (β 0.14), and a partner's favorable view on retiring early (β 0.15) were directly associated with an increased risk of early retirement, while not having a partner (β -0.13) was directly associated with a reduced risk of early retirement.	
18	Leijten 2015 (4)	The Netherlands	3	A sample of working individuals (STREAM)	45–64 years.<br><br>Mean age 53.4 ± 5.1 years	Both.<br><br>43.4% were women.	8149	Age, sex, physical load, job demands and job autonomy.<br><br>Educational level was grouped into low, medium, and high.	Self-reported early old-age retirement	HR 1.66 (95% CI 1.60–1.71) for 1-year increase in age, and 1.40 (95% CI 1.19–1.64) for male sex.<br><br>Estimated HR 0.7143 (95% CI 0.6084–0.8385) for women compared to men.	Age, sex, educational level, marital status, physical load, psychological job demands, lower autonomy, lower support, and chronic diseases (musculoskeletal,

No. of studies	First author and year of publication	Country	Follow-up time (years)	Study population	Age range at baseline	Sex	Sample size (in analysis)	Exposure	Retirement	Results	Adjustment for other covariates
								Physical workload was assessed using five items on force exertion, static load and vibration and dichotomized using the median.<br><br>Autonomy was assessed using five items, while psychological job demands and support from colleagues and/or supervisors were assessed using four items. Each of these items was based on a five-point Likert scale. There were dichotomized using the median.		HR 0.83 (95% CI 0.69–1.00) for low education and 0.85 (95% CI 0.72–1.02) for medium education compared with high level of education.<br><br>Estimated HR 0.841 (95% CI 0.740–0.954) for 12 years or less education compared to higher level.<br><br>HR 1.16 (95% CI 1.00–1.36) for high physical load, 0.98 (95% CI 0.84–1.14) for high psychological job demands, 1.08 (95% CI 0.93–1.25) for low autonomy, and 1.16 (95% CI 1.00–1.35) for low support.	severe headache, circulator, respiratory, digestive, diabetes and psychological)
18	de Wind 2014 (13)	The Netherlands	1 year	A sample of working individuals (STREAM)	59–63 years.<br><br>Mean age 60.7 years	Both.<br><br>41.6% were women.	2317	Age, sex, education, experiencing a severe life event, financial factors, physical workload, psychosocial factors, perceived physical and mental health, development of skills and knowledge, and enterprise restructuring.	Self-reported early old-age retirement.	OR 1.79 (95% CI 1.58–2.01) for 1-year increase in age.<br><br>OR 0.79 (95% CI 0.57–1.09) for women compared to men.<br><br>OR 0.92 (95% CI 0.60–1.40) for moderate physical health and 1.78 (95% CI 1.11–2.85) for poor physical health compared to good physical health.<br>OR 1.90 (95% CI 1.36–2.65) for poor	Age, sex, physical health, appreciation at work, skill and knowledge development, partner's attitude toward early retirement, and financial status

No. of studies	First author and year of publication	Country	Follow-up time (years)	Study population	Age range at baseline	Sex	Sample size (in analysis)	Exposure	Retirement	Results	Adjustment for other covariates
								Education was grouped into three levels: 1) low (primary school, lower and intermediate secondary education, or lower vocational training), 2) medium (higher secondary education, or intermediate vocational training), and 3) high (higher vocational education or university).<br><br>Perceived physical health was assessed using the physical component summary scale and perceived mental health was assessed using the mental component summary scale of the Short Form-12 Health Survey.<br><br>Physical workload was assessed using six items on force, using vibrating tools, awkward postures, prolonged standing, and		physical health compared to moderate or good physical health.<br><br>Estimated OR 1.235 (95% CI 0.902–1.693) for less than good physical health compared to good physical health.<br><br>OR 1.73 (95% CI 1.28–2.34) for lack of appreciation at work.<br><br>OR 2.24 (95% CI 1.60–3.13) for low vs. high development of skills and knowledge.<br><br>OR 3.01 (95% CI 2.22–4.08) for a positive attitude of the partner with respect to early retirement compared to negative or neutral attitude or not having a partner.<br><br>OR 6.78 (95% CI 4.56–10.09) for having the financial possibility to stop working before the retirement age compared to no or don’t know.<br><br>OR 1.24 (95% CI 0.95–1.62) for experiencing a severe life event (adjusted for age and sex).	

No. of studies	First author and year of publication	Country	Follow-up time (years)	Study population	Age range at baseline	Sex	Sample size (in analysis)	Exposure	Retirement	Results	Adjustment for other covariates
								prolonged squatting. Physical workload was classified into three groups using interquartile range.<br><br>Job demands, job autonomy and social support were assessed using four, five and four items, respectively.<br><br>Knowledge and skills development was measured using four items.		Unadjusted estimates. OR 0.83 (95% CI 0.60–1.34) for medium education and 1.03 (95% CI 0.76–1.40) for high education compared to low education.<br><br>OR 1.07 (95% CI 0.80–1.44) for medium and 1.13 (95% CI 0.81–1.58) for high physical workload compared to low physical workload.<br><br>Estimated OR 1.096 (95% CI 0.879–1.366) for moderate or high physical workload compared to low physical workload.<br><br>OR 0.93 (95% CI 0.78–1.10) for autonomy (a continuous variable, 1-unit increase).<br><br>OR 0.95 (95% CI 0.69–1.30) for moderate mental health and 0.92 (95% CI 0.63–1.33) for poor mental health compared to good mental health.<br><br>OR 1.05 (95% CI 0.77–1.42) for enterprise restructuring without compulsory redundancies and 0.75 (95% CI 0.48–1.17)	

No. of studies	First author and year of publication	Country	Follow-up time (years)	Study population	Age range at baseline	Sex	Sample size (in analysis)	Exposure	Retirement	Results	Adjustment for other covariates
										enterprise restructuring with compulsory redundancies.	
19	Reeuwijk 2017 (5)	11 European countries (SHARE study)	6	A random sample of general population	50-64 years.<br><br>Mean age 55.3 ± 3.6 years	Both.<br><br>43.8% were women.	5273	Perceived general health, assessed using a 5-point scale	Self-reported early old-age retirement at follow-up	Cause-specific Cox hazard ratio was 1.07 (95% CI 0.88-1.30) and Fine & Gray subdistribution hazard ratio was 0.94 (95% CI 0.77-1.16) for moderate or poor heath compared with good or excellent general health.	Sex, age, education, marital status, and welfare state regime
19	Kouwenhoven-Pasmooij 2016 (68)	11 European countries (SHARE study)	6	A random sample of general population	50-64 years.<br><br>Mean age 55.4 ± 3.6 years	Both.<br><br>44.9% were women.	5182	Self-reported stroke, heart disease and diabetes diagnosed by a physician	Self-reported early old-age retirement. at follow-up	OR 1.06 (95% CI 0.73–1.53) for diabetes, 1.61 (95% CI 1.15–2.27) for heart disease and 1.18 (95% CI 0.50–2.76) for stroke.	Age, sex, education, marital status, and European region
19	Robroek 2013 (3)	11 European countries (SHARE study)	4	A random sample of general population	50-64 years.<br><br>Mean age 55.2 ± 3.5 years	Both.<br><br>44% were women	4923	Perceived general health, assessed by using a 5-point scale.<br><br>BMI was based on self-reported height and weight grouped into normal (<25 kg/m^2), overweight, (≥25–<30 kg/m^2) and obese (≥30 kg/m^2).<br><br>Smoking was grouped into never, past, and current.	Self-reported early old-age retirement at follow-up	HR 1.17 (95% CI 0.90–1.52) for less than good perceived general health, 1.08 (95% CI 0.90–1.30) for overweight, 1.01 (95% CI 0.79–1.31) for obesity, 0.94 (95% CI 0.65–1.35) for lack of physical activity, and 1.31 (95% CI 1.06–1.63) for excessive alcohol use.<br><br>Estimated HR 1.0638 (95% CI 0.738–1.533) for moderate or vigorous physical activity.	Age, sex, education, marital status, perceived general health, BMI, lack of physical activity, excessive alcohol intake, low job control and low job rewards

No. of studies	First author and year of publication	Country	Follow-up time (years)	Study population	Age range at baseline	Sex	Sample size (in analysis)	Exposure	Retirement	Results	Adjustment for other covariates
								Lack of moderate or vigorous physical activity was based on self-reported activity less than once a week.<br><br>Excessive alcohol use was defined as intake of >2 glasses of alcohol beverages ≥5 days a week in the past 6 months.<br><br>Psychosocial factors at work were assessed by some items of the Job Content Questionnaire. Job control was assessed by two items and job rewards by five items. Physically demanding job and time pressure were assessed by a single 4-point scale.		HR 1.30 (95% CI 1.08–1.57) for low job control, 1.03 (95% CI 0.87–1.22) for low job rewards, 0.95 (95% CI 0.78–1.15) for past smoking, 0.93 (95% CI 0.75–1.14) for current smoking, 1.08 (95% CI 0.91–1.28) for physically demanding job, 1.00 (95% CI 0.85–1.18) for high time pressure, 1.25 (95% CI 0.99–1.58) for low job control in combination with high demands, and 0.99 (95% CI 0.83–1.18) for effort–reward imbalance.	
19	van den Berg 2010 (69)	11 European countries (SHARE study)	2 years	A random sample of general population	50–63 years	Both.<br><br>45% were women.	4611	Chronic disease, mobility problems and instrumental limitations.<br><br>Chronic diseases were included lifetime physician-diagnosed heart disease, stroke, diabetes, lung	Self-reported early old-age at follow-up	OR 1.28 (95% CI 1.01 to 1.62) for chronic disease.<br><br>OR 1.15 (95% CI 0.91 to 1.46) for mobility problems.<br><br>OR 0.99 (0.59 to 1.69) for instrumental limitations.	Age, sex, education, work characteristics, lifestyle factors and country

No. of studies	First author and year of publication	Country	Follow-up time (years)	Study population	Age range at baseline	Sex	Sample size (in analysis)	Exposure	Retirement	Results	Adjustment for other covariates
								disease, asthma, arthritis or rheumatism, and osteoporosis.<br><br>Mobility problems were characterized by the presence of one or more of 10 mobility problems, such as ability to walk.<br><br>Instrumental limitations referred to challenges in performing any of the 13 essential daily tasks, such as meal preparation, managing telephone calls.			
20	Morois 2016 (30)	France	Median follow-up 8.4 ± 3.7 years	Workers of a gas and electric company (GAZEL cohort study)	40–50 years	Men	8442	Alcohol consumption.<br><br>It was grouped into four levels: 1) low (1–40 g/day), 2) moderate (41–60 g/day), 3) high 61–100 g/day), and 4) very high (>100 g/day) consumption.	Register based early old-age retirement	Early old-age retirement (before age 55 years).<br><br>HR 1.14 (95% CI 1.04–1.26) for moderate drinking 1.20 (95% CI 1.09–1.32) for high drinking and 1.26 (95% CI 0.95–1.68) for very high drinking.<br><br>Early old-age retirement ($\geq$ age 55 years).	Age, socio-professional groups at hiring in 1989 and smoking status at baseline in 1991

No. of studies	First author and year of publication	Country	Follow-up time (years)	Study population	Age range at baseline	Sex	Sample size (in analysis)	Exposure	Retirement	Results	Adjustment for other covariates
										HR 1.10 (95% CI 1.02–1.19) for moderate drinking 0.99 (95% CI 0.91–1.07) for high drinking and 1.05 (95% CI 0.81–1.37) for very high drinking.<br><br>Estimated HR 1.046 (95% CI 0.991–1.105) for drinking more than 40 g/day.	
21	Friis 2007 (10)	Denmark	9 years	Register based nurses of the Danish Nurse Association	51–59 years	Women	5538	Place of residence, marital status, spouse's socioeconomic status, own gross income, working schedule, pressure of work/work speed, influence at work, physical demands at work, leisure-time physical activity, alcohol consumption. smoking, body mass index, and self-rated general health.<br><br>Register based data on place of residence, own annual gross income, marital status, and spouse's socioeconomic status.	Register based early old-age retirement	HR 1.19 (95% CI 1.10–1.29) for place of residence (outside of Copenhagen vs Copenhagen).<br><br>HR 0.89 (95% CI 0.80–0.98) for evening work, 1.04 (95% CI 0.92–1.19) for night work, and 0.80 (95% CI 0.72–0.89) for other shift works compared to day work.<br><br>HR 1.28 (95% CI 1.16–1.42) for annual own gross income of 200,000–249,999 Danish krone, 1.60 (95% CI 1.43–1.79) for 150,000–199,999 Danish krone, and 1.29 (95% CI 1.12–1.48) for less than 150,000 compared to 250,000 or more annual gross income.<br><br>Compared to workers without a spouse, HR	Place of residence, working schedule, working area, marital status and spouse's socioeconomic status, own gross income, pressure of work/work speed, influence at work, physical demands at work, body mass index, leisure-time physical activity, and self-rated general health

No. of studies	First author and year of publication	Country	Follow-up time (years)	Study population	Age range at baseline	Sex	Sample size (in analysis)	Exposure	Retirement	Results	Adjustment for other covariates
								Other exposures were assessed using a single question for each factor.		1.19 (95% CI 1.06–1.35) for employed spouse earning gross income of 400,000 Danish krone or more, 1.26 (95% CI 1.15–1.39) for those with a spouse earning less than 400,000, 1.64 (95% CI 1.49–1.80) for workers with a retired spouse, and 1.62 (95% CI 1.38–1.91) for those with an unemployed spouse.<br><br>HR 1.09 (95% CI 1.01–1.17) for high pressure of work/work speed compared to suitable or low.<br><br>HR 1.09 (95% CI 1.01–1.18) for some to low influence compared to high influence at work.<br><br>HR 1.08 (95% CI 1.00–1.17) for the presence of physical demands at work.<br><br>HR 1.13 (95% CI 1.04–1.22) for individuals who were physically active compared to those who were less active or inactive.<br><br>Estimated HR 0.885 (95% CI 0.817–0.958) for lack of moderate or	

No. of studies	First author and year of publication	Country	Follow-up time (years)	Study population	Age range at baseline	Sex	Sample size (in analysis)	Exposure	Retirement	Results	Adjustment for other covariates
										vigorous physical activity.<br><br>HR 0.97 (95% CI 0.89–1.07) for 1–4 drinks, 1.04 (95% CI 0.94–1.14) for 5–9 drinks, and 1.01 (95% CI 0.89–1.15) for 10 drinks or more compared to zero drinks.<br><br>Estimated HR 1.029 (95% CI 0.953–1.111) for drinking more than 4 drinks.<br><br>HR 0.98 (95% CI 0.90–1.06) for past smokers and 0.97 (95% CI 0.90–1.01) for never smokers compared to current smokers.<br><br>Estimated HR 1.01 (95% CI 0.9123–1.0745) for past smokers and 1.03 (95% CI 0.9165–1.0285) for current smokers compared to never smokers.<br><br>HR 1.12 (95% CI 1.04–1.21) for overweight (BMI 25–30 kg/m^2) and 1.06 (95% CI 0.91–1.24) for obesity (BMI >30 kg/m^2) compared to BMI <25 kg/m^2).	

No. of studies	First author and year of publication	Country	Follow-up time (years)	Study population	Age range at baseline	Sex	Sample size (in analysis)	Exposure	Retirement	Results	Adjustment for other covariates
										HR 1.28 (95% CI 1.16–1.41) for suboptimal (fair, poor or very poor) self-rated health compared to very good or good self-rated health.	
22	Blekesaune 2005 (71)	Norway	Mean follow-up 3.4 years (ranged from 1 to 7 years)	8.3% of the 1990 Norwegian population based on the census data consisted of Norwegian Social Science Data Services and Statistics Norway.	60–66 years	Both.<br><br>42.1% were women.	19 114	Age, marital status, education, income, physical job strains, low job autonomy and stressful job.<br><br>Register based education (ranged from 7 to 18 years) and income (ranged from 0 to 10) were analyzed as continuous variables.<br><br>Self-reported physical strains were assessed using three items and self-reported low job autonomy and stressful job were assessed each using two items. There variables were analyzed as scales ranging from 0 to 4.	Register based early old-age retirement	Men.<br>Logit coefficient 0.08 (SE 0.02) for 1-year increase in age.<br><br>Logit coefficient 0.23 (SE 0.08) for never married, 0.10 (SE 0.10) for widowed, 0.00 (SE 0.07) for separated or divorced, and -0.26 (SE 0.15) for cohabiting.<br><br>Logit coefficient -0.07 (SE 0.01) for each year increase in education, 0.06 (SE 0.02) for income and -0.02 (SE 0.00) for income squared.<br><br>Logit coefficient -0.06 (SE 0.03) for 1-unit increase in physical strain, -0.11 (SE 0.05) for 1-unit increase in stressful job, and 0.19 (SE 0.03) for 1-unit increase in low job autonomy.<br><br>Women.<br>Logit coefficient 0.19 (SE 0.02) for 1-year increase in age.	Age, marital status, education, income, physical job strains, job autonomy, stressful job, and calendar years.

No. of studies	First author and year of publication	Country	Follow-up time (years)	Study population	Age range at baseline	Sex	Sample size (in analysis)	Exposure	Retirement	Results	Adjustment for other covariates
										Logit coefficient -0.08 (SE 0.09) for never married, -0.28 (SE 0.06) for widowed, -0.42 (SE 0.08) for separated or divorced, and 0.30 (SE 0.18) for cohabiting.<br><br>Logit coefficient -0.02 (SE 0.01) for each year increase in education, -0.01 (SE 0.02) for income and -0.03 (SE 0.01) for income squared.<br><br>Logit coefficient 0.05 (SE 0.04) for 1-unit increase in physical strain, -0.10 (SE 0.06) for 1-unit increase in stressful job, and 0.03 (SE 0.03) for 1-unit increase in low job autonomy.<br><br>Both sexes combined. Estimated OR 1.145 (95% CI 1.113–1.177) for 1-year increase in age.<br><br>Estimated OR 0.900 (95% CI 0.846–0.957) for never married, widowed, or separated/divorced compared to married.	
23	Karpansalo 2005 (70)	Finland	Up to 15 years (average 11 years)	A representative sample of men living in the city of Kuopio and surrounding	42–60 years.	Men	1726	Depressive symptoms.	Register based non-illness early old-age retirement	HR 1.04 (95% CI 0.78–1.37) for the second tertile and 1.86 (95% CI 1.37–2.51) for the third tertile	Age, education, occupation, body mass index, alcohol consumption, smoking, maximal

No. of studies	First author and year of publication	Country	Follow-up time (years)	Study population	Age range at baseline	Sex	Sample size (in analysis)	Exposure	Retirement	Results	Adjustment for other covariates
				rural communities (the Kuopio ischemic heart disease risk factor study)	Mean age 51.8 ± 5.0 years			Depressive symptoms were assessed using the Human Population Laboratory depression scale. The tertile distribution was used for the analysis.		compared to the first tertile of depressive symptoms.<br><br>Estimated HR 1.362 (95% CI 1.108– 1.674) for the second or third tertile (depressive symptoms, yes/no).	oxygen uptake, and chronic diseases (musculoskeletal disorders, cardiovascular diseases, mental disorders, and other chronic illnesses)
23	Karpansalo 2004 (26)	Finland	Up to 15 years (average 11 years)	A representative sample of men living in the city of Kuopio and surrounding rural communities (the Kuopio ischemic heart disease risk factor study)	42–60 years.<br><br>Mean age 51.8 ± 5.0 years	Men	1748	Self-rated general health.<br><br>It was assessed using a single item and classified into three groups: very good or pretty good, 2) average, 3) pretty poor or very poor.	Register based non-illness early old-age retirement	HR 1.11 (95% CI 0.86–1.44) for average health and 3.36 (95% CI 2.20–5.13) for poor health compared to good self-rated health.<br><br>Estimated HR 1.498 (95% CI 1.202–1.866) for less than good general health.	Age, education, occupation, body mass index, alcohol consumption, smoking, maximal oxygen uptake, and chronic diseases (cardiovascular diseases, musculoskeletal disorders, and mental disorders)

References

1. Kadefors R, Nilsson K, Östergren PO, Rylander L, Albin M. Social inequality in working life expectancy in Sweden. Z Gerontol Geriatr 2019 Feb;52 Suppl 1:52–61. https://doi.org/10.1007/s00391-018-01474-3.
2. OECD. Flexible retirement in OECD countries, in Pensions at a Glance 2017: OECD and G20 Indicators, OECD Publishing, Paris, https://doi.org/10.1787/pension_glance-2017-5-en. 2017.
3. Robroek SJ, Schuring M, Croezen S, Stattin M, Burdorf A. Poor health, unhealthy behaviors, and unfavorable work characteristics influence pathways of exit from paid employment among older workers in Europe: a four year follow-up study. Scand J Work Environ Health 2013 Mar;39(2):125–33. https://doi.org/10.5271/sjweh.3319.
4. Leijten FR, de Wind A, van den Heuvel SG, Ybema JF, van der Beek AJ, Robroek SJ et al. The influence of chronic health problems and work-related factors on loss of paid employment among older workers. J Epidemiol Community Health 2015 Nov;69(11):1058–65. https://doi.org/10.1136/jech-2015-205719.
5. Reeuwijk KG, van Klaveren D, van Rijn RM, Burdorf A, Robroek SJ. The influence of poor health on competing exit routes from paid employment among older workers in 11 European countries. Scand J Work Environ Health 2017 Jan;43(1):24–33. https://doi.org/10.5271/sjweh.3601.
6. Risk Factors Collaborators GB; GBD 2021 Risk Factors Collaborators. Global burden and strength of evidence for 88 risk factors in 204 countries and 811 subnational locations, 1990-2021: a systematic analysis for the Global Burden of Disease Study 2021. Lancet 2024 May;403(10440):2162–203. https://doi.org/10.1016/S0140-6736(24)00933-4.
7. GBD 2021 Diseases and Injuries Collaborators. Global incidence, prevalence, years lived with disability (YLDs), disability-adjusted life-years (DALYs), and healthy life expectancy (HALE) for 371 diseases and injuries in 204 countries and territories and 811 subnational locations, 1990-2021: a systematic analysis for the Global Burden of Disease Study 2021. Lancet 2024 May;403(10440):2133–61. https://doi.org/10.1016/S0140-6736(24)00757-8.
8. Runge K, van Zon SK, Henkens K, Bültmann U. Metabolic syndrome and poor self-rated health as risk factors for premature employment exit: a longitudinal study among 55 016 middle-aged and older workers from the Lifelines Cohort Study and Biobank. Eur J Public Health 2024 Apr;34(2):309–15. https://doi.org/10.1093/eurpub/ckad219.

9. Breinegaard N, Jensen JH, Bonde JP. Organizational change, psychosocial work environment, and non-disability early retirement: a prospective study among senior public employees. Scand J Work Environ Health 2017 May;43(3):234–40. https://doi.org/10.5271/sjweh.3624.
10. Friis K, Ekholm O, Hundrup YA, Obel EB, Grønbaek M. Influence of health, lifestyle, working conditions, and sociodemography on early retirement among nurses: the Danish Nurse Cohort Study. Scand J Public Health 2007;35(1):23–30. https://doi.org/10.1080/14034940600777278.
11. Jacobsen PA, Kragholm K, Andersen MP, Lindgren FL, Ringgren KB, Torp-Pedersen C et al. Voluntary early retirement and mortality in patients with and without chronic diseases: a nationwide Danish Registry study. Public Health 2022 Oct;211:114–21. https://doi.org/10.1016/j.puhe.2022.07.019.
12. Wind A, Burr H, Pohrt A, Hasselhorn HM, Van der Beek AJ, Rugulies R. The association of health and voluntary early retirement pension and the modifying effect of quality of supervision: results from a Danish register-based follow-up study. Scand J Public Health 2017b Jul;45(5):468–75. https://doi.org/10.1177/1403494817699998.
13. de Wind A, Geuskens GA, Ybema JF, Blatter BM, Burdorf A, Bongers PM et al. Health, job characteristics, skills, and social and financial factors in relation to early retirement--results from a longitudinal study in the Netherlands. Scand J Work Environ Health 2014 Mar;40(2):186–94. https://doi.org/10.5271/sjweh.3393.
14. Page MJ, McKenzie JE, Bossuyt PM, et al. The PRISMA 2020 statement: an updated guideline for reporting systematic reviews. *BMJ* 2021;372 doi: ARTN n71
15. Shiri R, Poutanen J, Härmä M, Ervasti J, Haukka E. A meta-analysis of unemployment risk factors for middle-aged workers. Scand J Work Environ Health 2025 May;51(3):135–45. https://doi.org/10.5271/sjweh.4216.
16. Knardahl S, Johannessen HA, Sterud T, Härmä M, Rugulies R, Seitsamo J et al. The contribution from psychological, social, and organizational work factors to risk of disability retirement: a systematic review with meta-analyses. BMC Public Health 2017 Feb;17(1):176. https://doi.org/10.1186/s12889-017-4059-4.
17. Amiri S, Behnezhad S. Smoking and disability pension: a systematic review and meta-analysis. Public Health 2020 Sep;186:297–303. https://doi.org/10.1016/j.puhe.2020.04.013.
18. Shiri R, Falah-Hassani K, Lallukka T. Body mass index and the risk of disability retirement: a systematic review and meta-analysis. Occup Environ Med 2020 Jan;77(1):48–55. https://doi.org/10.1136/oemed-2019-105876.

19. Robroek SJ, Reeuwijk KG, Hillier FC, Bambra CL, van Rijn RM, Burdorf A. The contribution of overweight, obesity, and lack of physical activity to exit from paid employment: a meta-analysis. Scand J Work Environ Health 2013 May;39(3):233–40. https://doi.org/10.5271/sjweh.3354.
20. van Rijn RM, Robroek SJ, Brouwer S, Burdorf A. Influence of poor health on exit from paid employment: a systematic review. Occup Environ Med 2014 Apr;71(4):295–301. https://doi.org/10.1136/oemed-2013-101591.
21. Amiri S, Behnezhad S. Depression and risk of disability pension: A systematic review and meta-analysis. Int J Psychiatry Med 2019 May:91217419837412. https://doi.org/10.1177/0091217419837412.
22. Neovius K, Johansson K, Rössner S, Neovius M. Disability pension, employment and obesity status: a systematic review. Obes Rev 2008 Nov;9(6):572–81. https://doi.org/10.1111/j.1467-789X.2008.00502.x.
23. Carlsson E, Hemmingsson T, Landberg J, Burström B, Thern E. Do early life factors explain the educational differences in early labour market exit? A register-based cohort study. BMC Public Health 2023 Aug;23(1):1680. https://doi.org/10.1186/s12889-023-16626-3.
24. de Wind A, Geuskens GA, Ybema JF, Bongers PM, van der Beek AJ. The role of ability, motivation, and opportunity to work in the transition from work to early retirement--testing and optimizing the Early Retirement Model. Scand J Work Environ Health 2015 Jan;41(1):24–35. https://doi.org/10.5271/sjweh.3468.
25. Shiri R, El-Metwally A, Sallinen M, Pöyry M, Härmä M, Toppinen-Tanner S. The Role of Continuing Professional Training or Development in Maintaining Current Employment: A Systematic Review. Healthcare (Basel) 2023 Nov;11(21):2900. https://doi.org/10.3390/healthcare11212900.
26. Karpansalo M, Manninen P, Kauhanen J, Lakka TA, Salonen JT. Perceived health as a predictor of early retirement. Scand J Work Environ Health 2004 Aug;30(4):287–92. https://doi.org/10.5271/sjweh.796.
27. Almroth M, Falkstedt D, Hemmingsson T, Albin M, Badarin K, Selander J et al. Labour market exit routes in high- and low-educated older workers before and after social insurance and retirement policy reforms in Sweden. Ageing and Society. 2025;45(6):1228–47. https://doi.org/10.1017/S0144686X24000047.
28. Carlsson E, Hemmingsson T, Almroth M, Falkstedt D, Kjellberg K, Thern E. Mediating effect of working conditions on the association between education and early labour market exit: a cohort study of Swedish men. Occup Environ Med 2024 Dec;81(11):547–55. https://doi.org/10.1136/oemed-2024-109594.

29. Carlsson E, Hemmingsson T, Landberg J, Burström B, Thern E. The contribution of common mental disorders and alcohol-related morbidity to educational differences in early labour market exit among older workers: a register-based cohort study. Eur J Public Health 2025 Feb;35(1):65–71. https://doi.org/10.1093/eurpub/ckae212.

30. Morois S, Lemogne C, Leclerc A, Limosin F, Goldberg S, Goldberg M et al. More than Light Alcohol Consumption Predicts Early Cessation from Employment in French Middle-Aged Men. Alcohol Alcohol 2016 Mar;51(2):224–31. https://doi.org/10.1093/alcalc/agv092.

31. Sundstrup E, Hansen AM, Mortensen EL, Poulsen OM, Clausen T, Rugulies R et al. Retrospectively assessed physical work environment during working life and risk of sickness absence and labour market exit among older workers. Occup Environ Med 2018 Feb;75(2):114–23. https://doi.org/10.1136/oemed-2016-104279.

32. Hansen TH, Vignes B. Early retirement from the labour market among immigrants and natives: A register-based study of Norway. Nordic Welfare Research. 2022;7(2):75–95. https://doi.org/10.18261/nwr.7.2.1.

33. Harber-Aschan L, Chen WH, McAllister A, Koitzsch Jensen N, Thielen K, Andersen I et al. The impact of longstanding illness and common mental disorder on competing employment exits routes in older working age: A longitudinal data-linkage study in Sweden. PLoS One 2020 Feb;15(2):e0229221. https://doi.org/10.1371/journal.pone.0229221.

34. Higgins JP, Green S, editors. Cochrane Handbook for Systematic Reviews of Interventions Version 5.1.0 [updated March 2011]. The Cochrane Collaboration, 2011. Available from: www.handbook.cochrane.org.

35. Rice NE, Lang IA, Henley W, Melzer D. Common health predictors of early retirement: findings from the English Longitudinal Study of Ageing. Age Ageing 2011 Jan;40(1):54–61. https://doi.org/10.1093/ageing/afq153.

36. Gong CH, He X. Factors Predicting Voluntary and Involuntary Workforce Transitions at Mature Ages: evidence from HILDA in Australia. Int J Environ Res Public Health 2019 Oct;16(19):3769. https://doi.org/10.3390/ijerph16193769.

37. Dong L, Agnew J, Mojtabai R, Surkan PJ, Spira AP. Insomnia as a predictor of job exit among middle-aged and older adults: results from the Health and Retirement Study. J Epidemiol Community Health 2017 Aug;71(8):750–7. https://doi.org/10.1136/jech-2016-208630.

38. Olesen SC, Butterworth P, Rodgers B. Is poor mental health a risk factor for retirement? Findings from a longitudinal population survey. Soc Psychiatry Psychiatr Epidemiol 2012 May;47(5):735–44. https://doi.org/10.1007/s00127-011-0375-7.

39. Sundstrup E, Thorsen SV, Rugulies R, Larsen M, Thomassen K, Andersen LL. Importance of the Working Environment for Early Retirement: Prospective Cohort Study with

Register Follow-Up. Int J Environ Res Public Health 2021 Sep;18(18):9817. https://doi.org/10.3390/ijerph18189817.
40. Takada M, Tabuchi T, Iso H. Newly diagnosed disease and job loss: a nationwide longitudinal study among middle-aged Japanese. Occup Environ Med 2021 Apr;78(4):279–85. https://doi.org/10.1136/oemed-2020-106685.
41. Mein G, Martikainen P, Stansfeld SA, Brunner EJ, Fuhrer R, Marmot MG. Predictors of early retirement in British civil servants. Age Ageing 2000 Nov;29(6):529–36. https://doi.org/10.1093/ageing/29.6.529.
42. Pan T, Mercer SW, Zhao Y, McPake B, Desloge A, Atun R et al. The association between mental-physical multimorbidity and disability, work productivity, and social participation in China: a panel data analysis. BMC Public Health 2021 Feb;21(1):376. https://doi.org/10.1186/s12889-021-10414-7.
43. Mäcken J. Work stress among older employees in Germany: effects on health and retirement age. PLoS One 2019 Feb;14(2):e0211487. https://doi.org/10.1371/journal.pone.0211487.
44. Schinkel-Ivy A, Mosca I, Mansfield A. Factors Contributing to Unexpected Retirement and Unemployment in Adults Over 50 Years Old in Ireland. Gerontol Geriatr Med 2017 Jul;3:2333721417722709. https://doi.org/10.1177/2333721417722709.
45. Chen WH. Health and transitions into nonemployment and early retirement among older workers in Canada. Econ Hum Biol 2019 Dec;35:193–206. https://doi.org/10.1016/j.ehb.2019.06.001.
46. Houston DK, Cai J, Stevens J. Overweight and obesity in young and middle age and early retirement: the ARIC study. Obesity (Silver Spring) 2009 Jan;17(1):143–9. https://doi.org/10.1038/oby.2008.464.
47. Whittaker W, Higgerson J, Eden M, Payne K, Wilkie R, Verstappen SM. Effects of employees living with an 'arthritis' on sickness absence and transitions out of employment: a comparative observational study in the UK. RMD Open 2024 Nov;10(4):e004817. https://doi.org/10.1136/rmdopen-2024-004817.
48. Stynen D, Jansen NW, Kant I. The impact of work-related and personal resources on older workers' fatigue, work enjoyment and retirement intentions over time. Ergonomics 2017 Dec;60(12):1692–707. https://doi.org/10.1080/00140139.2017.1334094.
49. Nexo MA, Borg V, Sejbaek CS, Carneiro IG, Hjarsbech PU, Rugulies R. Depressive symptoms and early retirement intentions among Danish eldercare workers: cross-sectional and longitudinal analyses. BMC Public Health 2015 Jul;15:677. https://doi.org/10.1186/s12889-015-1973-1.

50. von Bonsdorff ME, Huuhtanen P, Tuomi K, Seitsamo J. Predictors of employees' early retirement intentions: an 11-year longitudinal study. Occup Med (Lond) 2010 Mar;60(2):94–100. https://doi.org/10.1093/occmed/kqp126.

51. Muurinen C, Laine M, Pentti J, Virtanen M, Salo P, Kivimäki M et al. Vertical and horizontal trust at work as predictors of retirement intentions: the Finnish Public Sector Study. PLoS One 2014 Sep;9(9):e106956. https://doi.org/10.1371/journal.pone.0106956.

52. Sejbaek CS, Nexo MA, Borg V. Work-related factors and early retirement intention: a study of the Danish eldercare sector. Eur J Public Health 2013 Aug;23(4):611–6. https://doi.org/10.1093/eurpub/cks117.

53. Bethge M, Radoschewski FM, Gutenbrunner C. The Work Ability Index as a screening tool to identify the need for rehabilitation: longitudinal findings from the Second German Sociomedical Panel of Employees. J Rehabil Med 2012 Nov;44(11):980–7. https://doi.org/10.2340/16501977-1063.

54. Szubert Z, Sobala W. Current determinants of early retirement among blue collar workers in Poland. Int J Occup Med Environ Health 2005;18(2):177–84.

55. Pan T, Mercer SW, Zhao Y, McPake B, Desloge A, Atun R et al. The association between mental-physical multimorbidity and disability, work productivity, and social participation in China: a panel data analysis. BMC Public Health 2021 Feb;21(1):376. https://doi.org/10.1186/s12889-021-10414-7.

56. Park J. Health factors and early retirement among older workers. Perspect Labour Income 2010;11(6):5–13.

57. Van Solinge H, Henkens K. Work-related factors as predictors in the retirement decision-making process of older workers in the Netherlands. Ageing Soc 2014;34(9):1551–74. https://doi.org/10.1017/S0144686X13000330.

58. Hale L, Singer L, Barnet JH, Peppard PE, Hagen EW. Associations Between Midlife Insomnia Symptoms and Earlier Retirement. Sleep Health 2017 Jun;3(3):170–7. https://doi.org/10.1016/j.sleh.2017.03.003.

59. Riekhoff AJ, Järnefelt N, Laaksonen M. Workforce Composition and the Risk of Labor Market Exit Among Older Workers in Finnish Companies. Work Aging Retire 2020;6(2):88–100. https://doi.org/10.1093/workar/waz023.

60. Runge K, van Zon SK, Henkens K, Bültmann U. Metabolic syndrome increases the risk for premature employment exit: A longitudinal study among 60 427 middle-aged and older workers from the Lifelines Cohort Study and Biobank. Scand J Work Environ Health 2023 Nov;49(8):569–77. https://doi.org/10.5271/sjweh.4113.

61. Jennen JG, Jansen NW, van Amelsvoort LG, Slangen JJ, Kant I. Chronic conditions and self-perceived health among older employees in relation to indicators of labour participation and retirement over time. Work 2022;71(1):133–50. https://doi.org/10.3233/WOR-210436.

62. De Breij S, Mäcken J, Qvist JY, Holman D, Hess M, Huisman M et al. Educational differences in the influence of health on early work exit among older workers. Occup Environ Med 2020 Aug;77(8):568–75. https://doi.org/10.1136/oemed-2019-106253.

63. Sewdas R, Thorsen SV, Boot CR, Bjørner JB, Van der Beek AJ. Determinants of voluntary early retirement for older workers with and without chronic diseases: A Danish prospective study. Scand J Public Health 2020 Mar;48(2):190–9. https://doi.org/10.1177/1403494819852787.

64. Thorsen SV, Jensen PH, Bjørner JB. Psychosocial work environment and retirement age: a prospective study of 1876 senior employees. Int Arch Occup Environ Health 2016 Aug;89(6):891–900. https://doi.org/10.1007/s00420-016-1125-7.

65. Oude Hengel K, Robroek SJ, Eekhout I, van der Beek AJ, Burdorf A. Educational inequalities in the impact of chronic diseases on exit from paid employment among older workers: a 7-year prospective study in the Netherlands. Occup Environ Med 2019 Oct;76(10):718–25. https://doi.org/10.1136/oemed-2019-105788.

66. Lund T, Villadsen E. Who retires early and why? Determinants of early retirement pension among Danish employees 57-62 years. Eur J Ageing 2005 Nov;2(4):275–80. https://doi.org/10.1007/s10433-005-0013-x.

67. de Wind A, Leijten FR, Hoekstra T, Geuskens GA, Burdorf A, van der Beek AJ. “Mental retirement?” Trajectories of work engagement preceding retirement among older workers. Scand J Work Environ Health 2017a Jan;43(1):34–41. https://doi.org/10.5271/sjweh.3604.

68. Kouwenhoven-Pasmooij TA, Burdorf A, Roos-Hesselink JW, Hunink MG, Robroek SJ. Cardiovascular disease, diabetes and early exit from paid employment in Europe; the impact of work-related factors. Int J Cardiol 2016 Jul;215:332–7. https://doi.org/10.1016/j.ijcard.2016.04.090.

69. van den Berg T, Schuring M, Avendano M, Mackenbach J, Burdorf A. The impact of ill health on exit from paid employment in Europe among older workers. Occup Environ Med 2010 Dec;67(12):845–52. https://doi.org/10.1136/oem.2009.051730.

70. Karpansalo M, Kauhanen J, Lakka TA, Manninen P, Kaplan GA, Salonen JT. Depression and early retirement: prospective population based study in middle aged men. J Epidemiol Community Health 2005 Jan;59(1):70–4. https://doi.org/10.1136/jech.2003.010702

71. Blekesaune M, Solem PE. Working conditions and early retirement - A prospective study of retirement behavior. Res Aging 2005;27(1):3–30. https://doi.org/10.1177/0164027504271438.
72. Stynen D, Jansen NW, Slangen JJ, de Grip A, Kant IJ. Need for recovery and different types of early labour force exit: a prospective cohort study among older workers. Int Arch Occup Environ Health 2019 Jul;92(5):683–97. https://doi.org/10.1007/s00420-019-01404-9.
73. Reeuwijk KG, de Wind A, Westerman MJ, Ybema JF, van der Beek AJ, Geuskens GA. 'All those things together made me retire': qualitative study on early retirement among Dutch employees. BMC Public Health 2013 May;13:516. https://doi.org/10.1186/1471-2458-13-516.
74. Eismann M, Henkens K, Kalmijn M. Why Singles Prefer to Retire Later. Res Aging 2019 Dec;41(10):936–60. https://doi.org/10.1177/0164027519873537.
75. Andersen LL, Thorsen SV, Larsen M, Sundstrup E, Boot CR, Rugulies R. Work factors facilitating working beyond state pension age: prospective cohort study with register follow-up. Scand J Work Environ Health 2021 Jan;47(1):15–21. https://doi.org/10.5271/sjweh.3904.
76. Burr H, Rauch A, Rose U, Tisch A, Tophoven S. Employment status, working conditions and depressive symptoms among German employees born in 1959 and 1965. Int Arch Occup Environ Health 2015 Aug;88(6):731–41. https://doi.org/10.1007/s00420-014-0999-5.
77. Burr H, Hasselhorn HM, Kersten N, Pohrt A, Rugulies R. Does age modify the association between psychosocial factors at work and deterioration of self-rated health? Scand J Work Environ Health 2017 Sep;43(5):465–74. https://doi.org/10.5271/sjweh.3648.
78. Toczek L, Peter R. Investigating the influence of work-related stress on early labour market exit: the role of health. Eur J Ageing 2023 Jul;20(1):31. https://doi.org/10.1007/s10433-023-00778-7.
79. Levinson D, Kaplan G. What does Self Rated Mental Health Represent. J Public Health Res 2014 Dec;3(3):287. https://doi.org/10.4081/jphr.2014.287.
80. Virtanen M, Oksanen T, Batty GD, Ala-Mursula L, Salo P, Elovainio M et al. Extending employment beyond the pensionable age: a cohort study of the influence of chronic diseases, health risk factors, and working conditions. PLoS One 2014 Feb;9(2):e88695. https://doi.org/10.1371/journal.pone.0088695.
